# Supplementary material for: Cobalt Borate Complex With Tetrahedrally Coordinated Co2+‐ Promotes Lithium Superoxide Formation in Li‐O2 Batteries
Source: Small. 2025 Jun 6;21(27):2502150. doi: 10.1002/smll.202502150 (PMC12243711; doi:10.1002/smll.202502150)
Supplement: Supplementary file 1 — Supporting Information [file SMLL-21-2502150-s001.docx]

Cobalt Borate Complex with Tetrahedrally Coordinated Co^2+^- Promotes Lithium Superoxide formation in Li-O_2_ Batteries

*Shivaraju Guddehalli Chandrappa,*^a,b,c,d^ Katrin Forster-Tonigold,^d,e^ Vasantha A. Gangadharappa,^a,b^Pavithra Kannan,^a,b^ Kunkanadu R Prakasha,^a,b^ Axel Groß,^d,f^ Maximilian Fichtner,^d,e^ Rachel A. Caruso,^c^ Guruprakash Karkera,^d^ and Annigere S. Prakash*^a,b^*

Shivaraju Guddehalli Chandrappa, Vasantha A. Gangadharappa, Pavithra Kannan

Kunkanadu R Prakasha Rachel A. Caruso, Annigere S. Prakash

CSIR - Central Electrochemical Research Institute-Chennai Unit, CSIR Madras Complex,

Taramani, Chennai-600113, India, Academy of Scientific and Innovative Research (AcSIR), Ghaziabad-201002, India, Applied Chemistry and Environmental Science, School of Science, RMIT University, Melbourne, Victoria 3000, Australia.

E-mail: [shivugc123@gmail.com](mailto:shivugc123@gmail.com), prakash.as@gmail.com

Shivaraju Guddehalli Chandrappa, Katrin Forster-Tonigold, Axel Groß, Maximilian Fichtner, Guruprakash Karkera

Helmholtz Institute Ulm for Electrochemical Energy Storage (HIU), 89081 Ulm, Germany, Institute of Nanotechnology, Karlsruhe Institute of Technology (KIT), P.O. Box 3640,

76021 Karlsruhe, Germany, Institute of Theoretical Chemistry, Ulm University, 89081 Ulm, Germany.

Keywords: oxygen evolution reaction, density functional theory, sodium cobalt borate, Li-O_2_ battery

The development of non-aqueous lithium-oxygen (Li-O_2_) batteries is hindered by inefficient discharge product decomposition, side reactions with the electrolyte, and high charge overpotentials (> 1 V). This study explores the use of sodium cobalt borate (Na_3_CoB_5_O_10_, NCBO) with cobalt in tetrahedral geometry as an oxygen electrocatalyst for non-aqueous Li-O_2_ batteries. The prepared cobalt borate exhibited an oxygen evolution reaction (OER) overpotential of 326 mV_RHE_ at a current density of 10 mA cm^-2^ and a Tafel slope of 42 mV dec^−1^ in 1 M KOH. Density Functional Theory (DFT) calculations identified the OH-covered (101) surface of NCBO as the preferred OER site, with an overpotential between 451-544 mV. In Li-O_2_ batteries, the NCBO cathode demonstrated 200 cycles with an overpotential of 1.95 V and 56.00 % round-trip efficiency at a capacity limit of 500 mA h g^-1^, along with a smaller charge overpotential of 0.64 V at a capacity limit of 2000 mA h g^-1^. Post-cycling analysis of NCBO electrodes revealed electronically conductive Lithium Superoxide (LiO_2_) as the dominant discharge product.

As revealed by DFT studies, the promising performance of NCBO in Li-O_2_ batteries is attributed to its tetrahedral Co coordination, highlighting its potential for electrocatalytic applications.

1. Introduction

Research on rechargeable Li-O_2_ batteries is presently being pursued due to its high theoretical specific energy density (∼3458 Wh kg^-1^), which is far beyond that of conventional Li-ion batteries^[1]^. However, Li-O_2_ batteries are still far from being commercialized because of a range of limitations, such as poor cyclability, low power density, low-rate capability, and large charge/discharge overpotentials. These are mainly attributed to the sluggish reaction kinetics of the O_2_ cathode during the lithium peroxide (Li_2_O_2_) formation (2Li^+^ + O_2_ → Li_2_O_2_, oxygen reduction reaction, ORR) and the Li_2_O_2_ decomposition (Li_2_O_2_ → 2Li^+^ + O_2_, oxygen evolution reaction, OER)^[2-3]^. The limited solubility and insulating nature of the Li_2_O_2_ discharge products that are deposited on the air cathode, gradually block the available surface area, resulting in poor electrochemical performance^[4-6]^. Thus, continuous efforts to construct new O_2_ cathode materials with improved electrochemical properties are needed to overcome these problems.

A new family of transition metal-based pentaborates Na_3_MB_5_O_10_ (where M = Co, Ni, Fe, etc.) has been explored for application as a Na-ion battery cathode material by Tarascon’s group^[7]^. The transition metal borates offer positive attributes in structural stability, especially in oxygen co-ordination of borate over conventional oxides, and can adopt different structural orientations like diborates [B_2_O_5_]^4-^, triborates [B_3_O_6_]^3-^, and pentaborates [B_5_O_10_]^5-[7]^. In addition, conventional Co based oxides have high symmetry octahedral co-ordination, whereas Co based compounds containing polyanion groups (borate, phosphate, sulphate, etc.) have low symmetry co-ordination, which include the tetrahedral (T_d_), trigonal planar, and trigonal bipyramidal crystal structure. Moreover, the presence of adaptable coordination of borate groups can potentially stabilize the intermediate state of metal active sites by changing their local crystal structure with ease, thus ensuring an efficient redox change in the transition metal^[7-9]^.

Recently, Baby *et al.*^[10]^ designed Zn-substituted cobalt phosphates for use as O_2_ cathode catalysts in Zn-air batteries, showing the ZnCoP_2_O_7_ catalyst to have enhanced Zn-air battery performance compared with conventional Co-based oxides. The authors attributed the enhanced activity to the presence of asymmetric CoO_5_/CoO_6_ polyhedra and edge-sharing between CoO_6_ octahedra and PO_4_^3-^. This allows the easy adsorption of the incoming O_2_/OH^-^ species on Co sites, thereby enhancing catalytic activity. Sharma *et al.*^[11]^ investigated fluorophosphate (Na_2_CoPO_4_F) as the O_2_ cathode catalyst for aqueous Na-air battery application. The presence of F in the structure modified the Co-O bond leading to higher catalytic activity. Kim *et al.*^[9]^ reported a series of cobalt polyphosphates for OER applications. They found that Na_2_CoP_2_O_7_ with low symmetry T_d_ geometry had excellent catalytic activity and stability that was comparable to conventional cobalt oxides. Dwibedi *et al.*^[12]^ reported alluaudite NaCoFe_2_(PO_4_)_3_ as a bi-functional catalyst, demonstrating that the inclusion of the phosphate group was attributed to the unique lattice structure geometry, facilitating the catalytic activity.

Solid Li_2_O_2_, with its electronically insulating nature, leads to higher overpotentials and adversely affects the long-term cycling stability of Li-O_2_ batteries^[13-14]^. In contrast, LiO_2_, formed via a one-electron transfer process, exhibits higher electronic conductivity and lower charge transfer resistance, thereby contributing to a reduced charge overpotential^[15-16]^. Therefore, recent research efforts have focused on developing suitable cathode catalysts that promote the dominant formation of LiO_2_ as the discharge product. However, to date, mainly iridium (Ir)-based catalysts have demonstrated the ability to facilitate LiO_2_ formation effectively^[15-20]^.

In this study, we investigated a transition-metal polyborate-based compound for OER and as an electrocatalyst in rechargeable Li-O_2_ batteries. To the best of our knowledge, transition-metal polyborates have not yet been explored as O_2_ cathode catalysts for Li-O_2_ batteries. This work studies the tetrahedrally coordinated Co^2+^ cobalt borate (NCBO) as the O_2_ catalyst in a Li-O_2_

battery. Post-cycling analysis of the NCBO electrodes revealed that electronically conductive LiO_2_ is the dominant discharge product.

## 2. Results and Discussion

The NCBO material was prepared by reacting sodium carbonate (Na_2_CO_3_), cobalt(II) hydroxide (Co(OH)_2_), and boric acid (H_3_BO_3_) in stoichiometric amounts under continuous inert Ar gas flow at 700 °C.

**Figure 1.** **(a)** Rietveld refinement of the XRD pattern of NCBO showing the experimental data points (red), calculated pattern (black continuous line), their differences (green line), and Bragg reflections (blue tick bars). **(b)** Crystal structure of NCBO and high-resolution XPS spectra of **(c)** Co 2p and **(d)** B 1s for NCBO.

**Figure 0.2.** Rietveld refinement of powder X-ray diffraction pattern of NCBO sample showing the experimental data points (red), calculated patterns (black continuous line), their differences (green line), and Bragg reflections (blue tick bars).

**Figure 0.3.** Rietveld refinement of powder X-ray diffraction pattern of NCBO sample showing the experimental data points (red), calculated patterns (black continuous line), their differences (green line), and Bragg reflections (blue tick bars).

**Figure 0.4.** Rietveld refinement of powder X-ray diffraction pattern of NCBO sample showing the experimental data points (red), calculated patterns (black continuous line), their differences (green line), and Bragg reflections (blue tick bars).

**Figure 0.5.** Rietveld refinement of powder X-ray diffraction pattern of NCBO sample showing the experimental data points (red), calculated patterns (black continuous line), their differences (green line), and Bragg reflections (blue tick bars).

**Figure 0.6.** Rietveld refinement of powder X-ray diffraction pattern of NCBO sample showing the experimental data points (red), calculated patterns (black continuous line), their differences (green line), and Bragg reflections (blue tick bars).

**Figure 0.7.** Rietveld refinement of powder X-ray diffraction pattern of NCBO sample showing the experimental data points (red), calculated patterns (black continuous line), their differences (green line), and Bragg reflections (blue tick bars).

**Figure 0.8.** Rietveld refinement of powder X-ray diffraction pattern of NCBO sample showing the experimental data points (red), calculated patterns (black continuous line), their differences (green line), and Bragg reflections (blue tick bars).

**Figure 0.9.** Rietveld refinement of powder X-ray diffraction pattern of NCBO sample showing the experimental data points (red), calculated patterns (black continuous line), their differences (green line), and Bragg reflections (blue tick bars).

**Figure 0.10.** Rietveld refinement of powder X-ray diffraction pattern of NCBO sample showing the experimental data points (red), calculated patterns (black continuous line), their differences (green line), and Bragg reflections (blue tick bars).

**Figure 0.11.** Rietveld refinement of powder X-ray diffraction pattern of NCBO sample showing the experimental data points (red), calculated patterns (black continuous line), their differences (green line), and Bragg reflections (blue tick bars).

**Figure 0.12.** Rietveld refinement of powder X-ray diffraction pattern of NCBO sample showing the experimental data points (red), calculated patterns (black continuous line), their differences (green line), and Bragg reflections (blue tick bars).

**Figure 0.13.** Rietveld refinement of powder X-ray diffraction pattern of NCBO sample showing the experimental data points (red), calculated patterns (black continuous line), their differences (green line), and Bragg reflections (blue tick bars).

**Figure 0.14.** Rietveld refinement of powder X-ray diffraction pattern of NCBO sample showing the experimental data points (red), calculated patterns (black continuous line), their differences (green line), and Bragg reflections (blue tick bars).

**Figure 0.15.** Rietveld refinement of powder X-ray diffraction pattern of NCBO sample showing the experimental data points (red), calculated patterns (black continuous line), their differences (green line), and Bragg reflections (blue tick bars).

**Figure 0.16.** Rietveld refinement of powder X-ray diffraction pattern of NCBO sample showing the experimental data points (red), calculated patterns (black continuous line), their differences (green line), and Bragg reflections (blue tick bars).

**Figure 0.17.** Rietveld refinement of powder X-ray diffraction pattern of NCBO sample showing the experimental data points (red), calculated patterns (black continuous line), their differences (green line), and Bragg reflections (blue tick bars).

**Figure 0.18.** Rietveld refinement of powder X-ray diffraction pattern of NCBO sample showing the experimental data points (red), calculated patterns (black continuous line), their differences (green line), and Bragg reflections (blue tick bars).

**Figure 0.19.** Rietveld refinement of powder X-ray diffraction pattern of NCBO sample showing the experimental data points (red), calculated patterns (black continuous line), their differences (green line), and Bragg reflections (blue tick bars).

**Figure 0.20.** Rietveld refinement of powder X-ray diffraction pattern of NCBO sample showing the experimental data points (red), calculated patterns (black continuous line), their differences (green line), and Bragg reflections (blue tick bars).

**Figure 0.21.** Rietveld refinement of powder X-ray diffraction pattern of NCBO sample showing the experimental data points (red), calculated patterns (black continuous line), their differences (green line), and Bragg reflections (blue tick bars).

**Figure 0.22.** Rietveld refinement of powder X-ray diffraction pattern of NCBO sample showing the experimental data points (red), calculated patterns (black continuous line), their differences (green line), and Bragg reflections (blue tick bars).

**Figure 0.23.** Rietveld refinement of powder X-ray diffraction pattern of NCBO sample showing the experimental data points (red), calculated patterns (black continuous line), their differences (green line), and Bragg reflections (blue tick bars).

**Figure 0.24.** Rietveld refinement of powder X-ray diffraction pattern of NCBO sample showing the experimental data points (red), calculated patterns (black continuous line), their differences (green line), and Bragg reflections (blue tick bars).

**Figure 0.25.** Rietveld refinement of powder X-ray diffraction pattern of NCBO sample showing the experimental data points (red), calculated patterns (black continuous line), their differences (green line), and Bragg reflections (blue tick bars).

**Figure 0.26.** Rietveld refinement of powder X-ray diffraction pattern of NCBO sample showing the experimental data points (red), calculated patterns (black continuous line), their differences (green line), and Bragg reflections (blue tick bars).

**Figure 0.27.** Rietveld refinement of powder X-ray diffraction pattern of NCBO sample showing the experimental data points (red), calculated patterns (black continuous line), their differences (green line), and Bragg reflections (blue tick bars).

**Figure 0.28.** Rietveld refinement of powder X-ray diffraction pattern of NCBO sample showing the experimental data points (red), calculated patterns (black continuous line), their differences (green line), and Bragg reflections (blue tick bars).

**Figure 0.29.** Rietveld refinement of powder X-ray diffraction pattern of NCBO sample showing the experimental data points (red), calculated patterns (black continuous line), their differences (green line), and Bragg reflections (blue tick bars).

**Figure 0.30.** Rietveld refinement of powder X-ray diffraction pattern of NCBO sample showing the experimental data points (red), calculated patterns (black continuous line), their differences (green line), and Bragg reflections (blue tick bars).

**Figure 0.31.** Rietveld refinement of powder X-ray diffraction pattern of NCBO sample showing the experimental data points (red), calculated patterns (black continuous line), their differences (green line), and Bragg reflections (blue tick bars).

**Figure 0.32.** Rietveld refinement of powder X-ray diffraction pattern of NCBO sample showing the experimental data points (red), calculated patterns (black continuous line), their differences (green line), and Bragg reflections (blue tick bars).


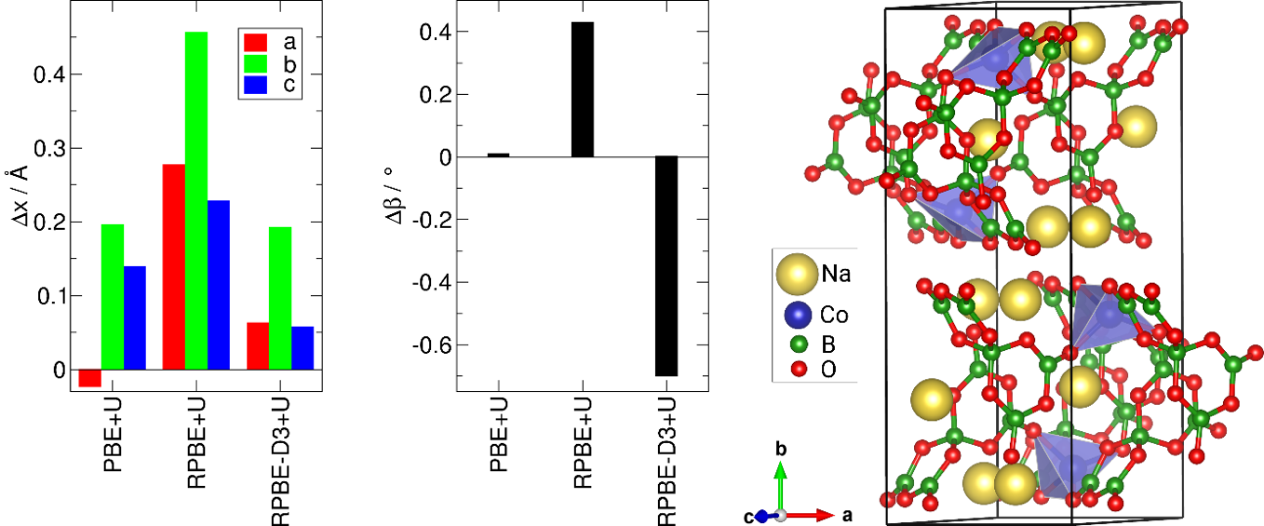


(b)

**Figure 1(a)** shows the Rietveld refinement of the powder x-ray diffraction (XRD) pattern of NCBO with a goodness of fit, Χ^2^ = 2.93. The XRD pattern of the NCBO can be indexed to a monoclinic structure with the space group *P2_1_/n* and lattice parameters: *a* = 6.6448(7) Å, b = 18.2061(2) Å, *c* = 7.8091(8) Å, α = γ = 90°, β = 114.81°, The corresponding structural parameters determined by Rietveld refinement are listed in **Table S1.**

All atoms in the prepared borate compound are placed in general positions 4e with one crystallographic site for cobalt, three for sodium, five for boron and ten for oxygen. The final refined atomic positions are given in **Table S1.** These results are in good agreement with other reported tetrahedral Co^2+^ coordinated compounds^[7]^. The corresponding crystal structure of NCBO is shown in **Figure 1(b).**

To estimate the oxidation state of elements in the prepared NCBO, X-ray photoelectron spectroscopy (XPS) studies were carried out. The survey spectrum reveals the coexistence of Na, Co, B, and O elements in NCBO (**Figure S1(a)**). As can be seen in **Figure 1(c)**, the Co 2p_3/2_ and Co 2p_1/2_ doublet in the Co 2p spectrum with binding energy values of 779.80 and 796.80 eV, respectively, correlate to the presence of Co^2+^ in NCBO^[21]^. The profile fitting of B 1s (**Figure 1(d)**) shows two peaks at ∼188 and ∼191 eV, which confirm the presence of B-Co and B-O bonds^[21]^. The O 1s spectrum shows a peak at ∼531.7 eV, indicating the presence of the O-B bond^[22]^ (**Figure S1(b)**).

The morphology and crystal structure of NCBO was examined on the nanoscale by transmission electron microscopy (TEM) **(Figure 2)**. The particles appeared agglomerated and in the size range of 50-200 nm, **Figure 2(a-b)**. The selected area electron diffraction (SAED) ring pattern (**Figure 2(c)**) confirms the polycrystalline nature of the NCBO. The SAED rings were indexed to the (043) and (-371) crystal planes of the NCBO crystal structure, corresponding to d-spacings of 0.20 and 0.16 nm, supporting the XRD findings. Furthermore, energy-dispersive X-ray spectroscopy (EDX) analysis in scanning transmission electron microscopy (STEM) mode **(Figures 2(d-h))** confirms the homogeneous distribution of Na, Co, B, and O elements throughout the as-synthesized powder.

The electrocatalytic activity of the borate compound towards oxygen evolution was investigated with linear sweep voltammetry (LSV) studies in N_2_ saturated 1 M KOH electrolyte at a scan rate of 5 mV s^-1^ and 1600 rpm. As shown in **Figure 3(a)**, NCBO has an overpotential of 326 mV_RHE_ and is comparable to the commercial RuO_2_ catalyst with an overpotential of 318 mV_RHE_ at an OER current density of 10 mA cm^-2^. To acquire more information on the kinetics of OER, the Tafel plots of NCBO and RuO_2_ catalysts are shown in **Figure 3(b)**, giving slopes of 42 and 82 mV dec^−1^, respectively, indicating faster OER kinetics of the borate catalyst. More insights into the OER mechanism in the aqueous, alkaline electrolyte system are given following the density functional studies in the next subsection. To probe the long-term OER stability of the NCBO catalyst, a chronopotentiometry (CP) test was performed at a fixed current density. As seen from [**Figure**](https://pubs.acs.org/doi/suppl/10.1021/acsaem.1c03103/suppl_file/ae1c03103_si_001.pdf) **S2,** the potential of the NCBO catalyst during the CP test at current density of 10 mA cm^–2^ remains quite stable even after 30 h.

**Figure 2.** **(a,b)** TEM images and (c) SAED pattern of NCBO. **(d)** High-angle annular dark-field (HAADF) image and STEM-EDX elemental mapping of (e) Na, (f) Co, (g) B, and (h) O in NCBO.

**Figure 0.33.** Rietveld refinement of powder X-ray diffraction pattern of NCBO sample showing the experimental data points (red), calculated patterns (black continuous line), their differences (green line), and Bragg reflections (blue tick bars).

**Figure 0.34.** Rietveld refinement of powder X-ray diffraction pattern of NCBO sample showing the experimental data points (red), calculated patterns (black continuous line), their differences (green line), and Bragg reflections (blue tick bars).

**Figure 0.35.** Rietveld refinement of powder X-ray diffraction pattern of NCBO sample showing the experimental data points (red), calculated patterns (black continuous line), their differences (green line), and Bragg reflections (blue tick bars).

**Figure 0.36.** Rietveld refinement of powder X-ray diffraction pattern of NCBO sample showing the experimental data points (red), calculated patterns (black continuous line), their differences (green line), and Bragg reflections (blue tick bars).

**Figure 0.37.** Rietveld refinement of powder X-ray diffraction pattern of NCBO sample showing the experimental data points (red), calculated patterns (black continuous line), their differences (green line), and Bragg reflections (blue tick bars).

**Figure 0.38.** Rietveld refinement of powder X-ray diffraction pattern of NCBO sample showing the experimental data points (red), calculated patterns (black continuous line), their differences (green line), and Bragg reflections (blue tick bars).

**Figure 0.39.** Rietveld refinement of powder X-ray diffraction pattern of NCBO sample showing the experimental data points (red), calculated patterns (black continuous line), their differences (green line), and Bragg reflections (blue tick bars).

**Figure 0.40.** Rietveld refinement of powder X-ray diffraction pattern of NCBO sample showing the experimental data points (red), calculated patterns (black continuous line), their differences (green line), and Bragg reflections (blue tick bars).

**Figure 0.41.** Rietveld refinement of powder X-ray diffraction pattern of NCBO sample showing the experimental data points (red), calculated patterns (black continuous line), their differences (green line), and Bragg reflections (blue tick bars).

**Figure 0.42.** Rietveld refinement of powder X-ray diffraction pattern of NCBO sample showing the experimental data points (red), calculated patterns (black continuous line), their differences (green line), and Bragg reflections (blue tick bars).

**Figure 0.43.** Rietveld refinement of powder X-ray diffraction pattern of NCBO sample showing the experimental data points (red), calculated patterns (black continuous line), their differences (green line), and Bragg reflections (blue tick bars).

**Figure 0.44.** Rietveld refinement of powder X-ray diffraction pattern of NCBO sample showing the experimental data points (red), calculated patterns (black continuous line), their differences (green line), and Bragg reflections (blue tick bars).

**Figure 0.45.** Rietveld refinement of powder X-ray diffraction pattern of NCBO sample showing the experimental data points (red), calculated patterns (black continuous line), their differences (green line), and Bragg reflections (blue tick bars).

**Figure 0.46.** Rietveld refinement of powder X-ray diffraction pattern of NCBO sample showing the experimental data points (red), calculated patterns (black continuous line), their differences (green line), and Bragg reflections (blue tick bars).

**Figure 0.47.** Rietveld refinement of powder X-ray diffraction pattern of NCBO sample showing the experimental data points (red), calculated patterns (black continuous line), their differences (green line), and Bragg reflections (blue tick bars).

**Figure 0.48.** Rietveld refinement of powder X-ray diffraction pattern of NCBO sample showing the experimental data points (red), calculated patterns (black continuous line), their differences (green line), and Bragg reflections (blue tick bars).

**Figure 0.49.** Rietveld refinement of powder X-ray diffraction pattern of NCBO sample showing the experimental data points (red), calculated patterns (black continuous line), their differences (green line), and Bragg reflections (blue tick bars).

**Figure 0.50.** Rietveld refinement of powder X-ray diffraction pattern of NCBO sample showing the experimental data points (red), calculated patterns (black continuous line), their differences (green line), and Bragg reflections (blue tick bars).

**Figure 0.51.** Rietveld refinement of powder X-ray diffraction pattern of NCBO sample showing the experimental data points (red), calculated patterns (black continuous line), their differences (green line), and Bragg reflections (blue tick bars).

**Figure 0.52.** Rietveld refinement of powder X-ray diffraction pattern of NCBO sample showing the experimental data points (red), calculated patterns (black continuous line), their differences (green line), and Bragg reflections (blue tick bars).

**Figure 0.53.** Rietveld refinement of powder X-ray diffraction pattern of NCBO sample showing the experimental data points (red), calculated patterns (black continuous line), their differences (green line), and Bragg reflections (blue tick bars).

**Figure 0.54.** Rietveld refinement of powder X-ray diffraction pattern of NCBO sample showing the experimental data points (red), calculated patterns (black continuous line), their differences (green line), and Bragg reflections (blue tick bars).


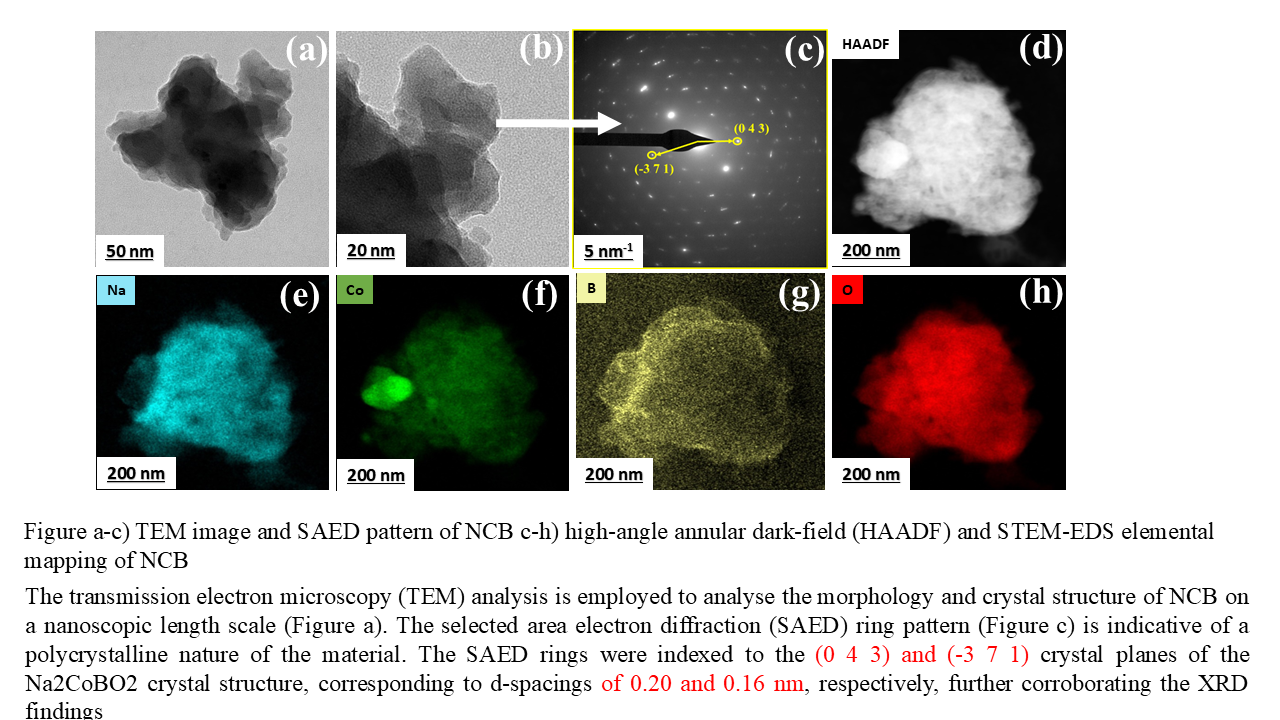


The electrocatalytic activity of the NCBO catalyst was also investigated in a nonaqueous electrolyte. **Figure 3(c)** presents the cyclic voltammograms (CV) of NCBO and Ketjen black (KB) at a scan rate of 0.1 mV s^-1^ between 2.0 and 4.5 V in 1 M lithium bis(trifluoromethanesulfonimide (LiTFSi) in tetraethylene glycol dimethyl ether (TEGDME). The cyclic voltammogram of the NCBO cathode electrode shows a cathodic peak at ∼2.5 V, which corresponds to the oxygen reduction or discharge reaction (formation of lithium peroxide (Li_2_O_2_): O_2_ + 2Li^+^ + 2e^-^ → Li_2_O_2_)^[23]^. The anodic peak at ∼3.5 V is ascribed to the oxygen evolution or charge reaction (decomposition of Li_2_O_2_: Li_2_O_2_ → O_2_ + 2Li^+^ + 2e^-^)^[24]^. The NCBO cathode exhibits an efficient oxygen reduction reaction (ORR) onset potential and higher OER/ORR peak current density than KB, implying that NCBO is more effective in the Li_2_O_2_ generation/decomposition. This enhanced performance is likely due to the superior electronic conductivity and catalytic activity of NCBO, which facilitate faster electron transfer and more efficient reactions compared to KB.

**Figure 3(d)** shows the specific capacity *vs.* voltage profile of the NCBO and KB with a capacity limitation of 2000 mA h g^-1^ at a current density of 175 mA g^-1^. The NCBO shows a smaller charge overpotential (0.64 V) than KB (1.04 V). This suggests that NCBO catalyzes the decomposition of the discharge products at a lower overpotential, making it more efficient for OER. The cycling stability of the Li-O_2_ batteries with the NCBO cathode was evaluated at a current density of 175 mA g^-1^ and at a capacity limitation of 500 mA h g^-1^ (**Figure 3(e))**. During the first cycle, the NCBO electrode shows a charge-discharge overpotential of 1.15 V with 70.51% round trip efficiency, after 200 cycles it shows an overpotential of 1.95 V with 56% round trip efficiency.

**Figure 3.** (a) iR-corrected LSV plots of OER and (b) Tafel plots of the NCBO and RuO_2_ catalysts in 1 M KOH electrolyte. (c) CV curves of NCBO and KB electrodes at a scan rate of 0.1 mV s^-1^ and between 2.0-4.5 V. (d) The discharge/charge profiles of the NCBO and KB electrodes at a current density of 175 mA g^-1^ and a cut-off capacity of 2000 mA h g^-1^. (e) Cyclic stability test of the NCBO O_2_ cathode (current density of 175 mA g^-1^, cut-off capacity of 500 mA h g^-1^). (f) Nyquist plots (inset: higher magnification of Nyquist plots) and (g) long cycle stability test for NCBO and KB electrodes at a current density of 175 mA g^-1^.


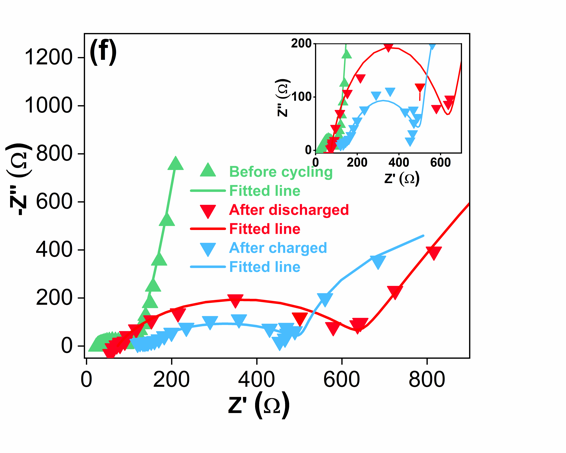

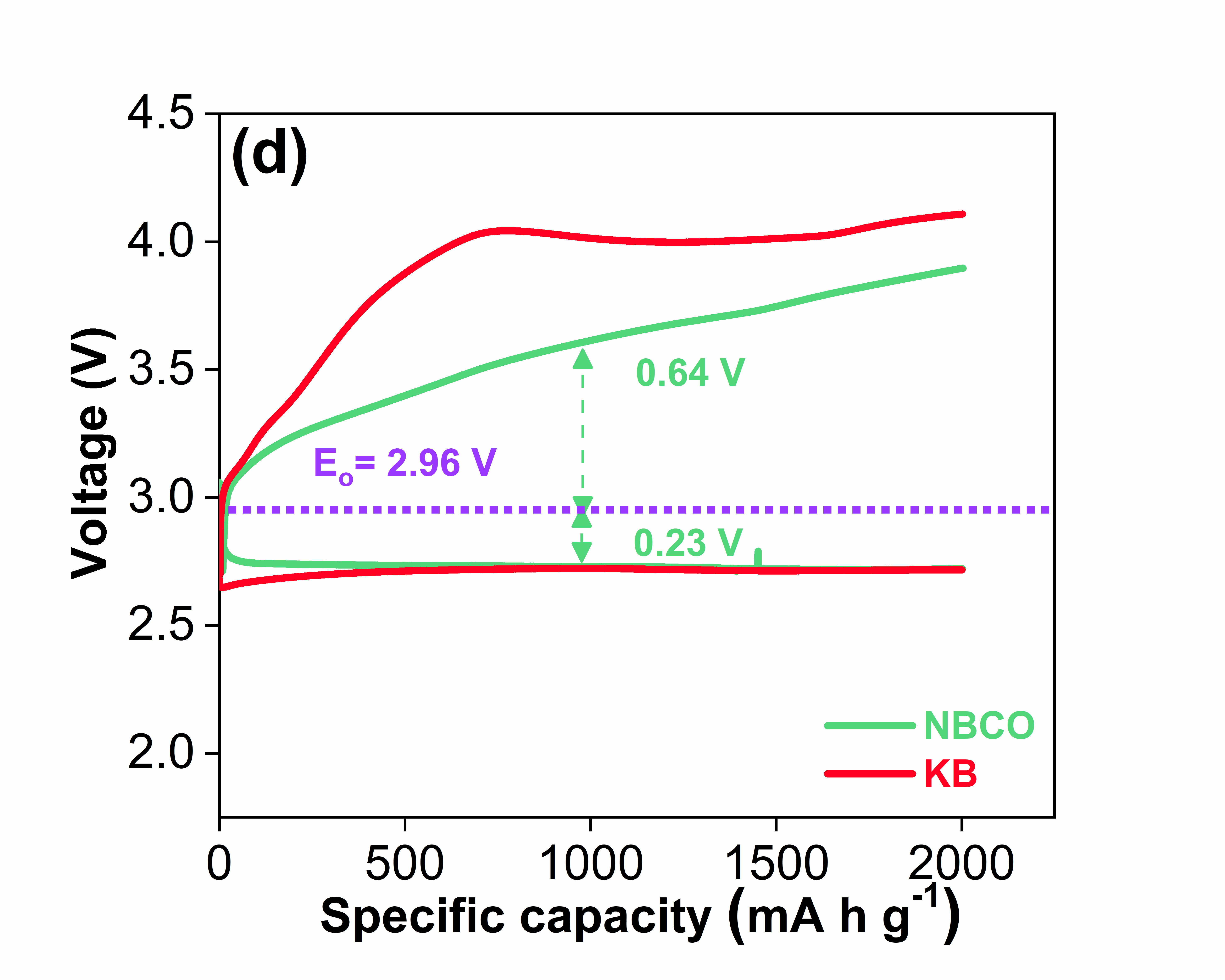

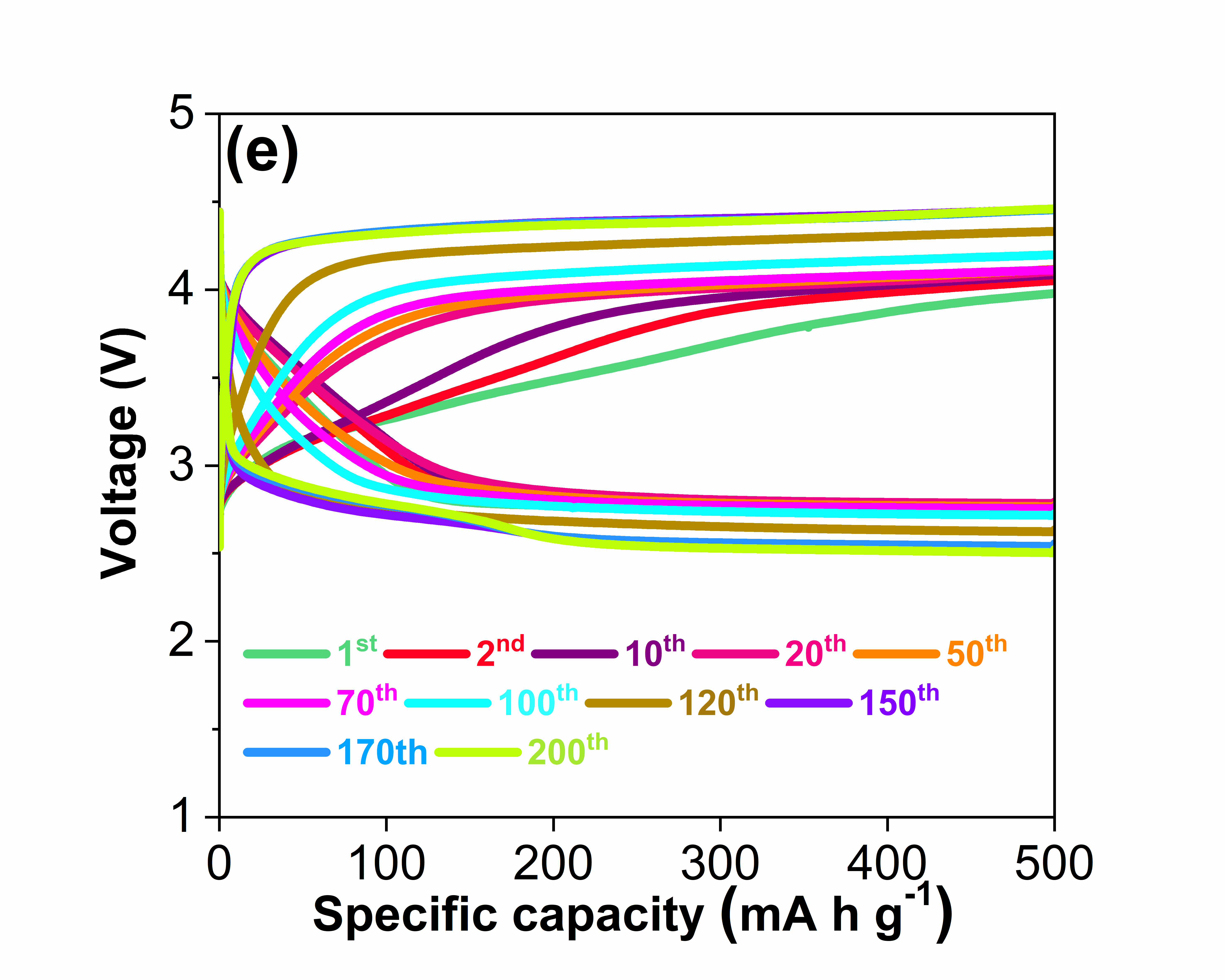

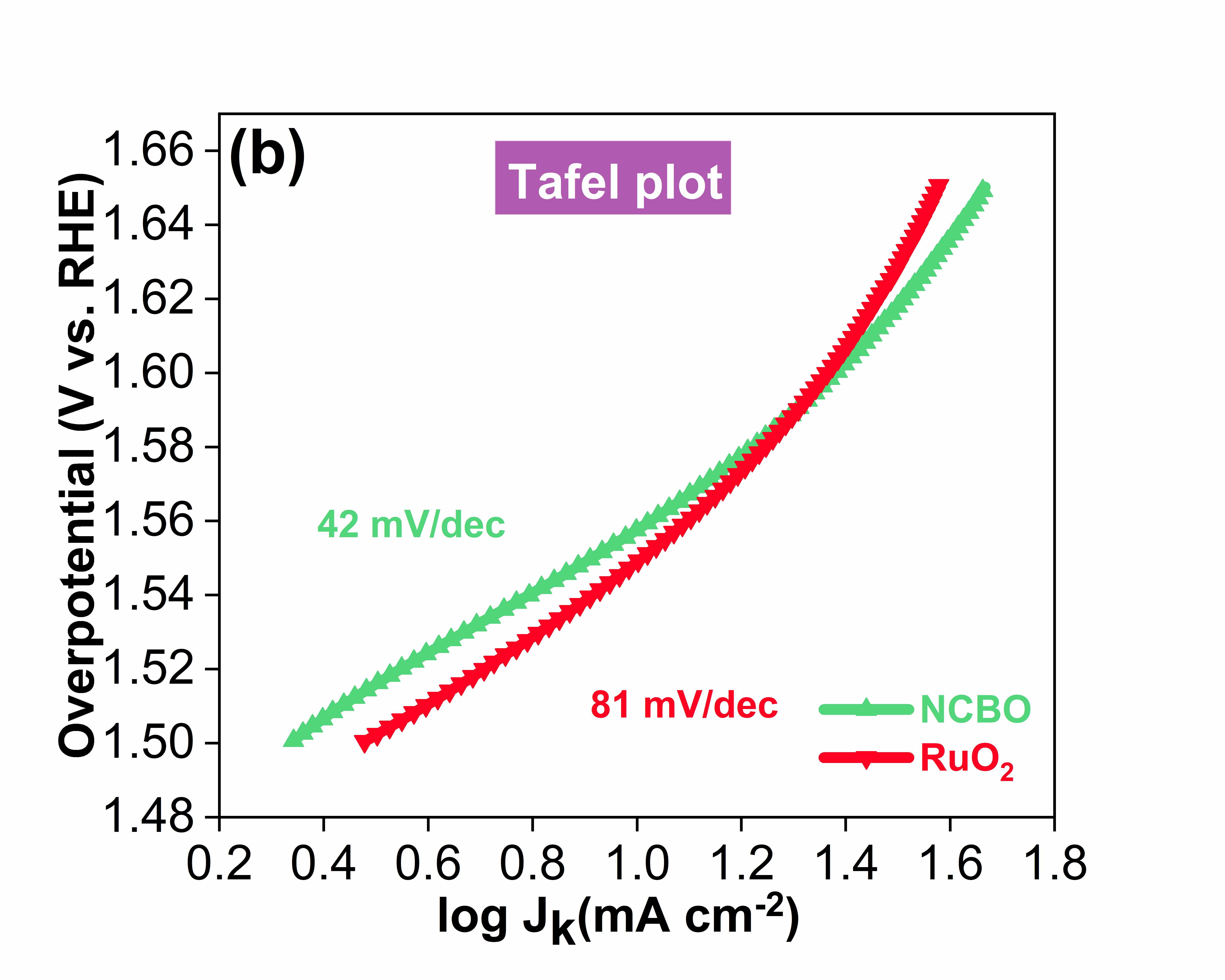

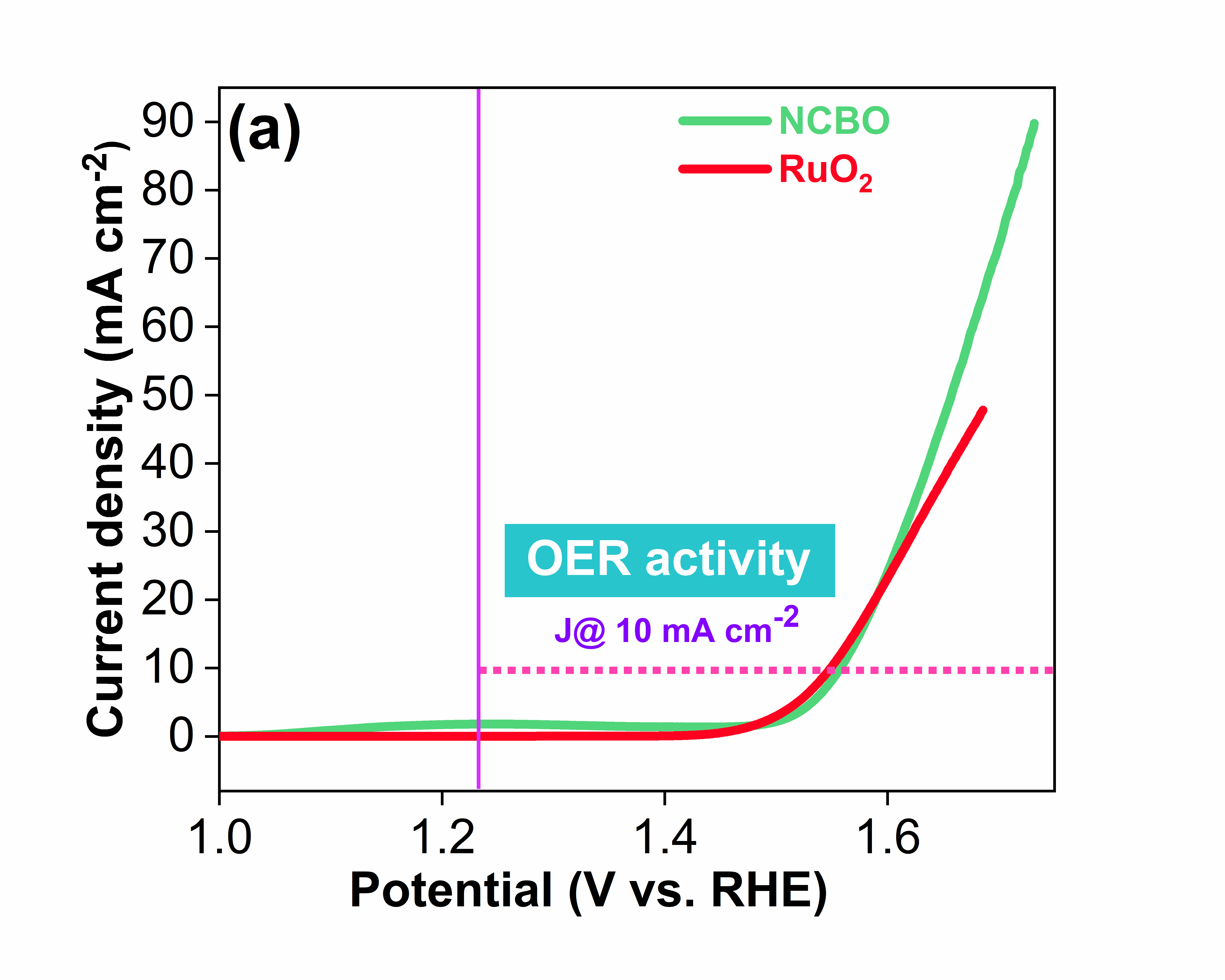

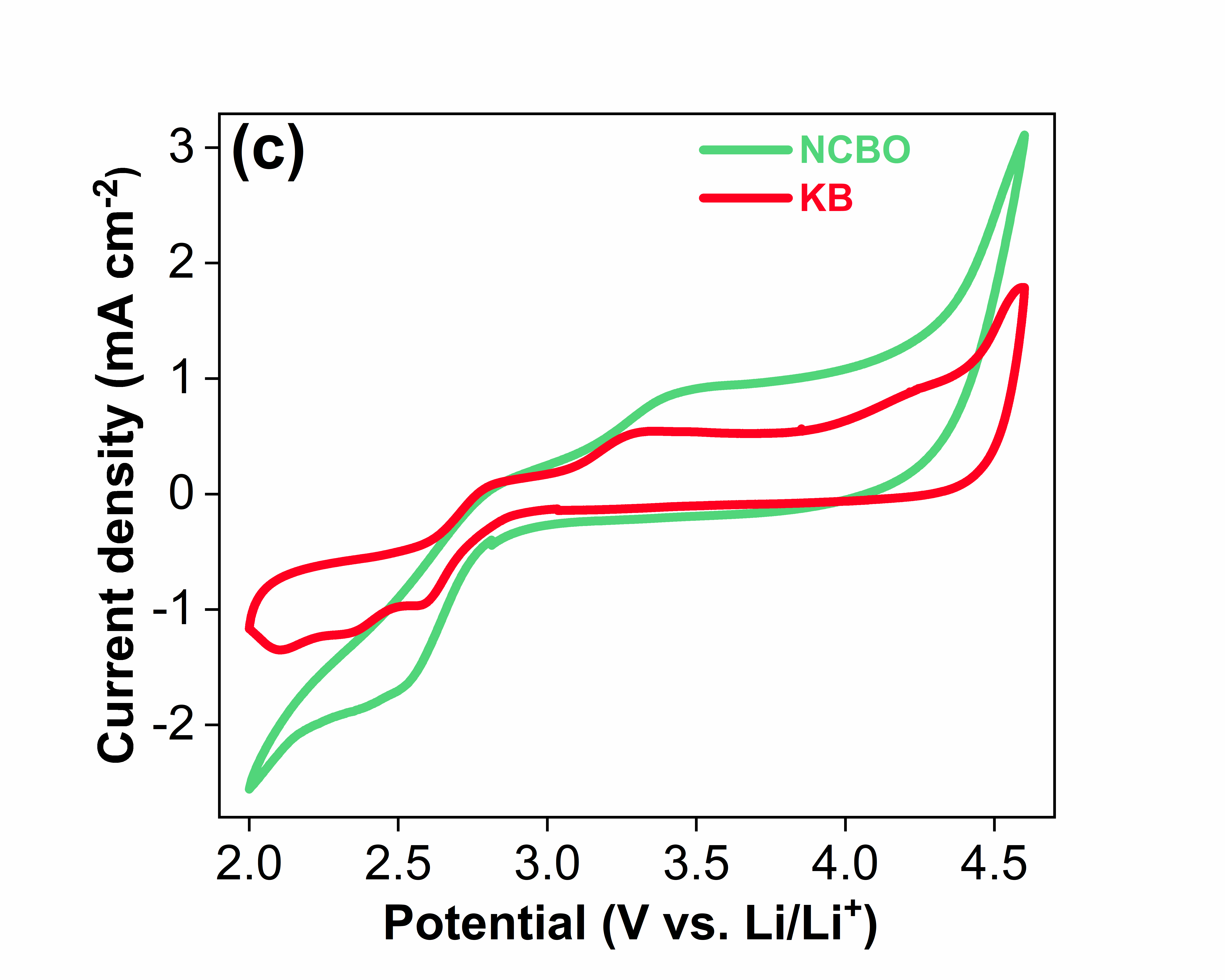


The charge transfer resistance (R_3_) during the discharge and charge process of the NCBO cathode containing Li-O_2_ cells was determined using electrochemical impedance spectroscopy (EIS). **Figure 3(f)** shows the Nyquist plots of the cell before cycling, after first discharge, and after first charge of the NCBO cathode. The EIS data have been simulated by Zsimpwin software using equivalent circuit R_1_(Q_1_R_2_)(Q_2_R_3_)W, where R_1_ is solution resistance, R_2_ is solid-electrolyte interface resistance, Q_1_ and Q_2_ are constant phase elements, and W is attributed to the mass transfer of O_2_ at the cathode catalyst. The simulated EIS results are given in **Table S2**. The R_3_ value of the pristine electrode is ∼70 Ω, which significantly increased to ∼559 Ω after the discharge, attributable to the accumulation of the insulting Li_2_O_2_ discharge product on the surface of the electrode. Upon charging, the R_3_ value decreases to ∼303 Ω, suggesting the decomposition of Li_2_O_2_ discharge product on the cathode surface.

The long cyclability of the NCBO cathode was investigated at a capacity limitation of 500 mA h g^-1^ and compared with KB. As shown in **Figure 3(g),** the assembled Li-O_2_ cell with the NCBO catalyst shows a smaller charge-discharge voltage gap after 200 cycles and cycled over 1150 h, while for KB increased overpotentials are observed just after 200 h. This is speculated to result from the accumulation of other irreversible discharge products, primarily originating from reactions between the electrolyte, carbon, and lithium at higher charge voltage (>4.5 V), rather than Li_2_O_2_, on the cathode surface.

Furthermore, the morphology of transient intermediates formed during the charge/discharge of Li-O_2_ batteries was investigated using ex-situ Field Emission scanning electron microscope (FE-SEM) analysis. **Fig. 4(a-h)** presents the FE-SEM images of pristine, discharged, and charged electrodes. The discharged electrode [**Fig 4(c-f)**] exhibits the formation of lower-like Li_2_O_2_/LiO_2_  structures on the surface of the NCBO cathode during the fully discharged state. These flower-like Li_2_O_2_/LiO_2_ structures completely disappear in the charged state, indicating their decomposition upon charging [**Fig 4(g-h)**].

In addition, ex-situ XRD **(Fig.4(i))** patterns of pristine, discharged, and charged electrodes were compared with the XRD pattern of commercial Li_2_O_2_. In the discharged cathode, the presence of the three diffraction peaks observed at 2θ = 32.9 and 35.0° which correspond to the Li_2_O_2_ phase in the.The weak intensity of these peaks is attributed to the dominant presence of highly crystalline carbon components (polyvinylidene difluoride (PVDF) and KB) in the sample. Interestingly, these reflections disappeared in the charged cathode, suggesting that the discharge products undergo reversible formation and decomposition during the electrochemical process^[25]^. In addition, no distinct diffraction peaks corresponding to LiO_2_ were observed in the XRD patterns, likely due to its amorphous nature^[26]^.

Similarly, ex-situ Raman spectroscopy (**(Fig.4(f))** revealed peaks at ∼1125 and ∼1516 cm⁻¹ in the discharged electrode, indicating the presence of lithium superoxide (LiO_2_). The peak at ∼1125 cm⁻¹ is characteristic of a LiO_2_-like species, whereas the peak at ∼1516 cm⁻¹ is associated with strong interactions between LiO_2_ and the graphite carbon surface. Additionally, a low intense peak corresponding to Li_2_O_2_ was also observed. The relative intensity of LiO_2_ was much higher than that of Li_2_O_2_, which indicated that LiO_2_ was the dominant discharge product. During the charge, the characteristic peaks of LiO_2_ and Li_2_O_2_ were disappeared, indicating their decomposition. Interestingly, catalysts that promote the formation of LiO_2_ have been shown to enhance battery performance and cycle life, mainly due to its higher electronic conductivity and lower charge transfer resistance, thereby contributing to a reduced charge overpotential^[13, 15, 26-28]^. The ex-situ XPS results of the cycled NCBO cathodes are provided in the Supporting Information **(Figure S3)**.


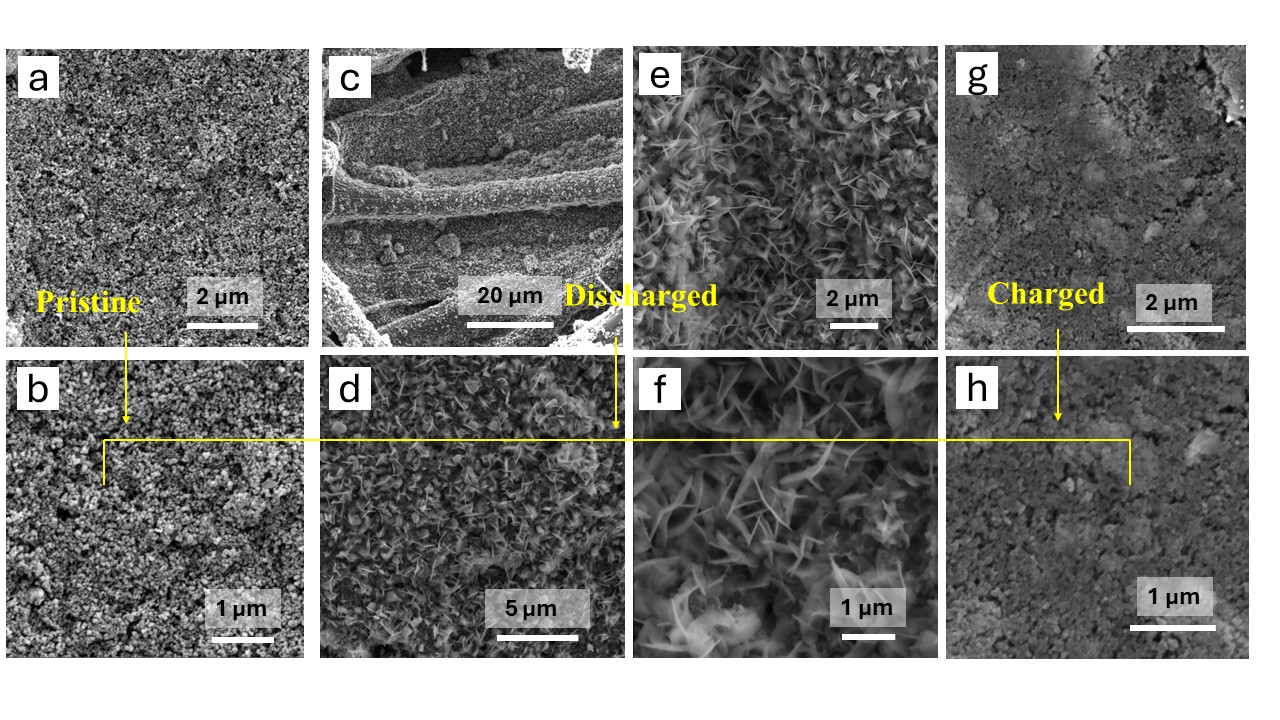


**Figure 4.** Ex-situ analysis of the NCBO cathode: FESEM images of (a,b)pristine, (c-f) discharged and (g,h) charged electrodes; (i) XRD patterns; and (j) Raman spectra of the pristine, discharged, and charged electrodes.

**2.1. Density functional theory calculations**

The OER mechanism in the aqueous, alkaline electrolyte system on NCBO was studied by periodic DFT calculations. We considered different surfaces of the NCBO crystal, the structure of which is well described by differently corrected generalized gradient approximation (GGA) functionals (see Supporting Information, **Figure S4** and **Table S3**). Surface energies were calculated employing the PBE+U method. The (010) surface **(see Figure 5a)** is the most stable surface, having a surface energy of 21.4 meVÅ^-2^. By cutting normal to the [010] direction, the tetrahedral coordination of the Co atoms is kept intact and only ionic bonds between Na atoms and O atoms of borate molecules are withdrawn. No pronounced relaxation effects occur. To create a (101) surface, not only Na-O bonds but also Co-O bonds need to be broken, leading to threefold coordinated surface Co atoms (see **Figure 5b**). If no ionic relaxation were allowed, this would be associated with an energy penalty of 53.3 meVÅ^-2^. However, due to a rearrangement of the threefold coordinated surface Co atoms towards neighbouring tetrahedral sites, a considerable energy gain is obtained. The resulting surface structure contains units of two corner sharing, distorted CoO_4_-tetrahedrons and has a surface energy of 31.9 meV Å^-2^. Other surfaces, such as the (001) and (011) surfaces, have higher surface energies (75.4 and 39.3 meV Å^-2^, respectively) and were not considered in the calculations of the OER mechanism.

Our calculations of the OER mechanism are based on the commonly employed assumption that the alkaline OER proceeds in four elementary steps, where * indicates the adsorption of intermediates onto the catalyst surface:

∗ + OH^−^ → ∗OH + e^−^ (1)
∗OH + OH^−^ → ∗O + H_2_O (l) + e^−^ (2)
∗O + OH^−^ → ∗OOH + e^−^ (3)
∗OOH + OH^−^ → ∗ + O_2_ (g) + H_2_O (l) + e^−^ (4)

leading to the total reaction as follows.

4OH^−^ → O_2_ (g) + 2H_2_O (l) + 4e^−^ (5)

We would like to note that the electronic structure of NCBO strongly depends on the strength of the on-site correction for highly correlated electrons (see Supporting Information, **Figure S5**), unlike the geometrical structure that depends merely on the actual value of the Hubbard correction (see Supporting Information, **Table S3).** This may impact the interaction of different adsorbates with the substrate to different degrees. Therefore, the OER mechanism has been studied by employing different values for the Hubbard correction. Further details on the calculations of the individual reaction energies and the overpotential are given in the Supporting Information, page 26-27**.**


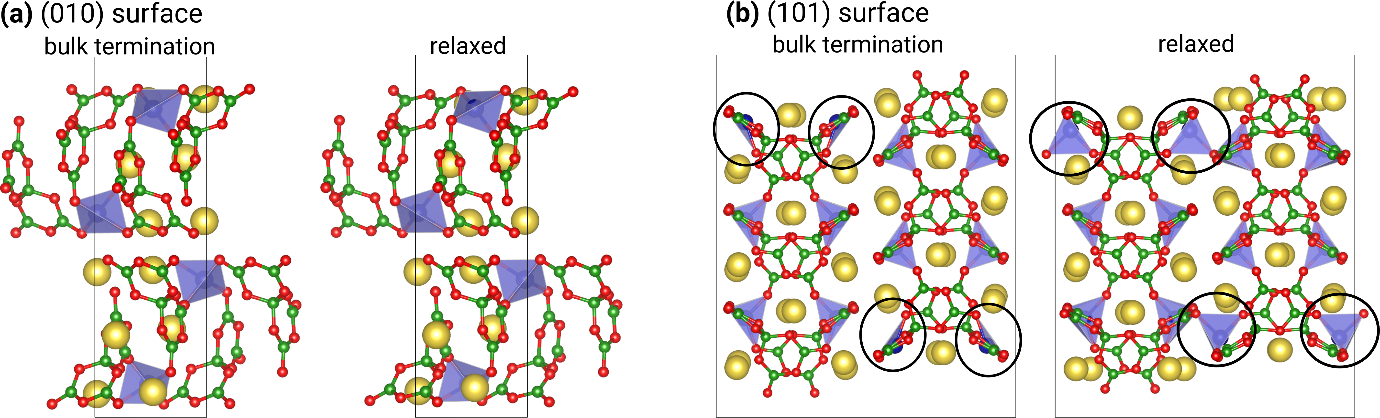


**Figure 5**. (a) The (010) surface and (b) the (101) surface slabs of NCBO before (left) and after (right) ionic relaxation (Color coding correlates to image in Figure 1(b)). In (b) black circles mark the strong relaxation of the threefold coordinated Co surface atoms leading to a tetrahedral coordination.

The reaction energies and structures of the intermediates of the OER at the (010) surface of NCBO is shown in **Figure 6a** and **6c**, respectively. The first reaction step involves the adsorption of OH. This leads to a change of the fourfold tetrahedral coordination of Co to a distorted fivefold bipyramidal coordination. The charge of Co, as calculated according to a Bader charge analysis, increases from +1.27 e for the bare surface to +1.59 e for the OH adsorption complex. This reflects the increase in the oxidation state of Co. Besides the bonding of OH to Co, Na surface atoms stabilize the OH adsorption. If the PBE+U method is used, the Na-O distances of the adsorption complex are 2.35 Å and 2.39 Å, which are close to the Na-O distances reported for NaOH (2.29 Å, 2.37 Å and 2.40 Å for space group P21/m^[29]^ as well as 2.31 Å and 2.38 Å for space group Cmcm)^[30]^

The following reaction from adsorbed OH to an adsorbed O atom (reaction step 2) is associated with the highest step in energy and thus represents the potential determining step (PDS). The Co-O distance clearly decreases from 1.99 Å to 1.64 Å, the charge on Co slightly diminishes to +1.50 e. The charge on Co remains at about +1.49 e after the formation of adsorbed OOH (reaction step 3). The adsorption complex of OOH includes a less distorted bipyramidal coordination of Co, with a Co-O distance of 1.97 Å. Finally, the release of O_2_ (reaction step 4) is an exergonic process at pH 13.

Applying a ‘+U’ correction of 3.5 eV to the pure or dispersion corrected GGA functionals, the structures of the intermediates of the alkaline OER mechanism as well as their thermodynamic energies are rather similar for the PBE and the RPBE-D3 functional. The overpotential is 0.76 eV for the PBE+U(3.5 eV) method and 0.74 eV for RPBE-D3+U(3.5 eV). At a lower coverage (0.25 molecules per surface unit cell, not shown), the overpotential decreases slightly to 0.74 V if the PBE+U(3.5 eV) method is applied.

**Figure 6**. (a,b) Total reaction energies $\text{∆G(}j\text{)}=\sum_{i}^{j} \text{∆}\text{G}_{i}$ (upper panels) and individual reaction energies $\text{∆}\text{G}_{i}$ (lower panels) for the alkaline OER reaction steps (pH=13) calculated (a) at the bare (010) and (b) at the partially OH-covered (101) surface of NCBO with different exchange correlation functionals and different values of the Hubbard-like correction term U. The structural models of the individual reaction steps at the (010) and partially OH-covered (101) surface are shown in subfigures (c) and (d), respectively. The color-coding of most of the atoms is given in Figure 1b, pink atoms reflect oxygen atoms involved in the OER, purple atoms denote the oxygen of the pre-adsorbed OH molecule, and white atoms are hydrogen.


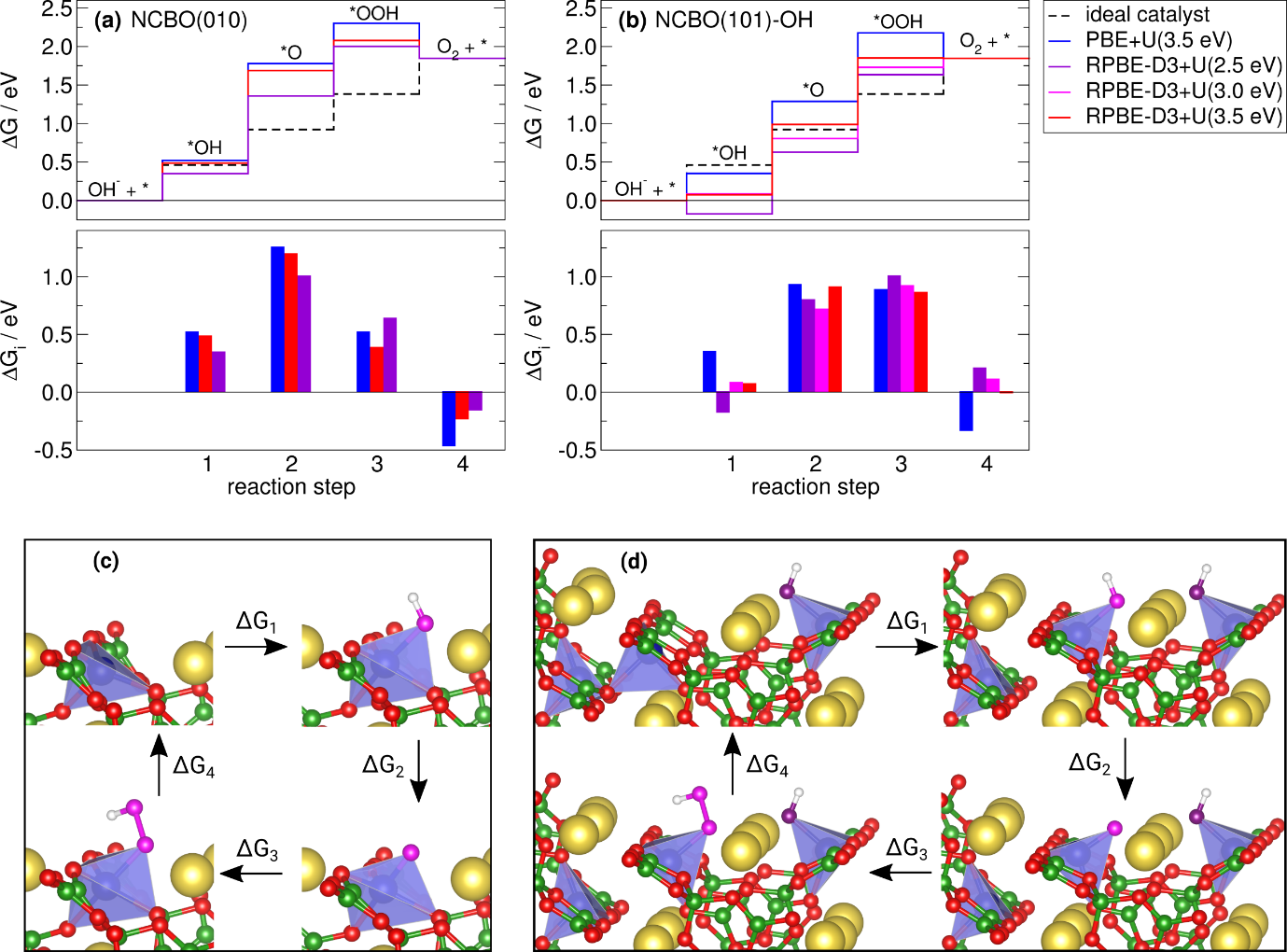


Yet, a remarkable decrease in the individual reaction energies ΔG_i_ is obtained if a smaller ‘+U’-parameter of 2.5 eV is employed, leading to an overpotential of 0.55 V.

The impact of the electronic correlation on the overpotential of the OER was already observed by García-Mota *et al.*, who also found a destabilization of the O, OH and OOH adsorbates on Co-oxide and -oxohydroxide surfaces due to the Hubbard correction, which is most pronounced for the adsorbed O-atom^[31]^. According to their RPBE+U(U=3.5 eV) calculations the overpotential of the OER is 0.76 V at a Co_3_O_4_ surface and 0.78 V at a CoOOH surface, which is about 0.35 V (Co_3_O_4_ surface) or 0.51 V (CoOOH surface) larger than the overpotential calculated with the uncorrected GGA functional RPBE^[32]^. Notably, though different coordination environments of Co are present, the overpotentials calculated at the Co-oxide surfaces are close to our calculated overpotentials at the (010) surface, if comparable methods are used. Additionally, the impact of explicit solvent molecules on the OER was studied by employing static models of the (010) surface that include one or six water molecules per unit cell (see Supporting Information, **Figure S6**). As previously, the second reaction step (*OH🡪 *O) represents the PDS. Yet, as the adsorption of OH is energetically less favorable in the presence of water molecules, the overpotential, calculated by the PBE+U method, decreases to 0.61 eV (for one co-adsorbed H_2_O per surface unit cell) or 0.63 eV (for 6 co-adsorbed H_2_O molecules per surface unit cell).

We observe an exergonic adsorption of OH, for the slightly less stable (101) surface of NCBO. The free energy of reaction step (1) at the bare surface is about -0.5 eV. This energy gain can be explained by the fact that upon OH adsorption, the surface relaxation effect, which leads to Co atoms coordinated within two corner-sharing tetrahedrons, is relieved. Instead, the original tetrahedral coordination of Co atoms in bulk is restored by replacing the missing borate-oxygen at the surface with OH (see purple OH in Figure 5d). Thus, at pH=13, the (101) surface is at least partially covered with OH, and to study the OER reaction mechanism at the (101) surface, co-adsorbed OH molecules need to be taken into account. The results of different calculations of the OER mechanism at such a surface are shown in **Figure 6b** and **6d**. The adsorption of the second OH molecule occurs at the second surface Co atom of the unit cell, leading to a tetrahedrally coordinated Co atom at the surface in the same fashion as already observed for the adsorption of the first OH. At the second reaction step, leading to the formation of an adsorbed O-atom, the tetrahedral coordination gets distorted: the adsorbed O atom is slightly displaced towards the surface Na atoms. The Na-O distances are 2.31 Å and 2.33 Å, suggesting that there are electrostatic Na-O interactions that stabilize the adsorbed O atom. The reaction energy of this step is much lower than the corresponding value at the (010) surface. Furthermore, it is comparable to the energy of the formation of adsorbed OOH for which the tetrahedral coordination is restored.

According to PBE+U calculations, the overpotential is associated with reaction step 2 as PDS and amounts to 0.47 V, which is 0.29 V lower than the overpotential at the (010) surface. If the RPBE-D3+U method is used, the overpotential amounts to 0.45, 0.46 or 0.54 V for U=3.5, 3.0 or 2.5 eV, respectively. For U ≤ 3.0 eV the PDS changes from reaction step 2 to reaction step 3.

Noteworthy and possibly also assignable to other GGA+U calculations of the OER at inorganic surfaces, is the fact that the nature of the PDS and the actual value of the overpotential both depend on the strength of the Hubbard correction applied on top of the GGA functional in order to cope with the strongly correlated d-electrons of Co.

3. Conclusion

A tetrahedrally coordinated cobalt borate (NCBO) material has been explored as an OER catalyst for rechargeable Li-O_2_ battery applications. The catalyst displayed an efficient OER performance in 1 M KOH solution with an OER overpotential of 326 mV_RHE_ at a current density of 10 mA cm^-2^ and a small Tafel slope of 42 mV dec^−1^. DFT calculations revealed a preferred OER at the OH-covered (101) surface of NCBO with an overpotential in the range of about 451-544 mV. The calculated overpotential is slightly larger than the measured one, however, regarding the simplifications and assumptions of the calculational setup, the computational values compare reasonably well to the experimentally determined value. The reaction steps in which the adsorbed O or OOH are formed might be both the PDS as they have almost equal reaction energies. A rechargeable Li-O_2_ battery was constructed using NCBO as an air cathode. The NCBO catalyst shows a smaller charge overpotential of 0.64 V at a capacity limitation of 2000 mA h g^-1^ and a current density of 175 mA g^-1^. The long-cycle stability of the NCBO cathode was evaluated at a limited capacity of 500 mA h g^-1^ for 200 cycles, delivering an overpotential of 1.95 V and a 56.00% round-trip efficiency. Post-mortem analysis of cycled NCBO electrodes revealed electronically conductive Lithium Superoxide as the dominant discharge product.

4. Experimental Section/Methods

*Materials:* The chemicals used for the NCBO synthesis were Na_2_CO_3,_ (Merck India, 99.5 %), (Co(OH)_2_, (Sigma-Aldrich, 95%), and H_3_BO_3_ (Merck India, 99.5 %).

For electrochemical activity tests, the following chemicals and materials were used: Potassium hydroxide (KOH, AR, 99 %, SRL, India), Nafion ionomer (Sigma Aldrich), ethanol (Merck India), Milli-Q water and The catalyst RuO_2_ (99.9% trace metals basis, Sigma Aldrich) was purchased for comparative studies.

For Li-O_2_ battery studies, the following chemicals and materials were utilized:The following chemicals and materials used for Li-O2 battery studies lithium disc (8 mm diameter, Alfa-Aesar), glass microfiber separator (Whatman, Sigma Aldrich), KB, (akzonobel), PVDF, (Sigma Aldrich), N-methyl-2-pyrrolidone (NMP, Sigma Aldrich), carbon paper (SIGRACET GDL 38 BC), TEGDME, (Sigma Aldrich) and highly pure oxygen (99.999% purity, INDO GAS).

*Material synthesis:* NCBO was prepared by a conventional ceramic method. All chemicals were dried in a vacuum oven at 70 °C for 24 h before use. Stoichiometric amounts of Na_2_CO_3_, Co(OH)_2_, and H_3_BO_3_ were ball milled for 40 min under argon using a high-energy milling apparatus (Fritsch P-23). The obtained solid was hand-ground and heated to 700 °C for 1 h in a tube furnace (Ants Ceramics, India) under argon flow. The prepared sample was stored in an Ar-filled glovebox until use to avoid oxidation by moist air.

*Material characterization:* XRD patterns of the prepared borate were measured on a Bruker D8 Advance Da Vinci diffractometer using Cu Kα (λ = 1.5418 Å) radiation. The oxidation state of the samples was determined from XPS using a Thermo Fischer K-Alpha™ Spectrometer with non-monochromatic Al Kα radiation (1486.7 eV). The C 1s peak of the adventitious carbon (284.8 eV) was used as the reference binding energy for calibration. The morphological features were observed on a Carl Zeiss 130 VP FESEM.

Raman spectra were taken on an TechnoS IndiRAM CTR 500C Micro Raman Spectrometer using a 532 nm diode laser. High-resolution transmission electron microscopy (HRTEM) images were obtained with a Thermo Scientific Talos F200S. The elemental mapping of the NCBO was obtained by Bruker EDX attached to HRTEM.

### *Electrochemical activity tests:* Rotating-disc electrode experiments were carried out in a PINE RDE setup with glassy carbon (5 mm in diameter, PINE Research Instrumentation, Inc. USA) as the working electrode, Pt wire as the counter electrode and Hg/HgO (1 M KOH) as the reference electrode. The catalyst ink was prepared by mixing 14 mg of catalyst and 4 mg carbon black in a mixture of 100 μL of Nafion ionomer, 600 μL of ethanol and 300 μL Milli-Q water. The 10 μL dispersed catalyst ink was cast on the glassy carbon (loading = 0.7 mg cm^-2^). The catalytic activity (OER) studies of the catalysts were carried out in both N_2_ and O_2_-saturated aqueous 1 M KOH electrolyte solutions.

The potentials recorded and referred to Hg/HgO in each experiment were calculated using the formula

E_RHE_ = E_Hg/HgO_ + 0.059 pH + 0.198 V (1)

where E_Reversible hydrogen electrode (RHE)_ is the potential vs. RHE, E_Hg/HgO_ is the potential vs. Hg/HgO electrode, and 1 M KOH accounts for a pH of 13.

The RuO_2_ catalyst with the same loading was prepared using same method for comparison.

### *Li-O_2_ battery fabrication and testing:* The Li-O_2_ battery studies of prepared catalysts were carried out in a Swagelok-type cell in an argon-filled glovebox. A lithium disc (8 mm) was used as the anode with a glass microfiber separator. The cathode disc was prepared by depositing the slurry mixture of catalyst, KB and PVDF in NMP on the carbon paper, with a catalyst: KB: PVDF ratio of 60:40:10). A 10 mm diameter coated gas diffusion layer (GDL) with a catalyst loading of ≈0.3-0.5 mg cm^-2^ was used as the cathode in the Li-O_2_ cell. The current density and specific capacity of the cathode were calculated based on the mass of the cathode catalyst. The specific capacity and current density were expressed in terms of the total mass of the cathode catalyst. 1 M LiTFSi in TEGDME was used as the electrolyte. Highly pure oxygen at a pressure of 1 bar was supplied from the direction of the cathode for cell reactions. The galvanostatic charge–discharge, cyclic voltammetry and electrochemical impedance spectroscopy (EIS) studies were conducted at room temperature using VMP3Z biologic multi-channel. For the EIS analysis, a frequency range of 10 mHz–1 MHz with an amplitude of 10 mV was used.

*Computational method:* We used the Vienna ab initio simulation package (VASP 5.4)^[33-34]^ to perform periodic density functional theory calculations. The electron-ion interaction was described by the projected augmented wave method^[35-36]^. The electronic wave functions were expanded in a plane wave basis set up to a cutoff energy of 500 eV. The generalized gradient approximation (GGA) was employed to calculate the exchange and correlation energy. In detail, mainly the PBE functional^[37]^ was used. For comparison, its revised version of Hammer and Nørskov (RPBE)^[38]^ in combination with the semi-empirical correction scheme for dispersive interactions of Grimme (D3)^[39]^ was consulted. The damping function proposed by Chai and Head-Gordon (“zero-damping”) was chosen to circumvent the divergence of the dispersion correction at short distances^[40]^. To account for on-site Coulomb interactions a Hubbard like term (+U) was added in the way proposed by Dudarev^[41]^.When the PBE or RPBE functional were used without dispersion correction, the values of U_eff_=U-J were set to U=3.5 eV and J=0 eV for the Co-atoms in NCBO, following the suggestions given for PBE^[42]^ and for RPBE^[31]^. In case of the dispersion corrected RPBE functional, we additionally employed a slightly different value of the Hubbard parameter (U=2.5 eV), as previously determined^[43]^.

All geometry optimizations were carried out until all forces on atoms were less than 0.01 eV^-^Å. The electronic structure was converged within 10^-6^ eV. For the integration over the first Brillouin zone a Gaussian smearing of 0.05 eV was used. Furthermore, calculations of bulk NCBO employed a 7x1x5 k-point mesh.

The NCBO surfaces were modeled by symmetric slabs consisting of formula units that were separated by a vacuum region of 15 Å. During geometry optimization the atoms in the middle of the (101) slab and the atoms in the lower half of the (010) slab were kept fixed to mimic the bulk structure. For calculations of the (010) and (101) surface a 7x1x5 and a 5x1x1 k-point mesh was employed, respectively.

**Supporting Information**

Supporting Information is available from the Wiley Online Library or from the author.

Acknowledgements

The authors gratefully acknowledge the Department of Science & Technology (DST), New Delhi, India for their financial support (Grant No.DST/CEST/AMEST/2022/65(G)) and thank the CSIR-CECRI for providing access to the characterization (CIF) facilities. CSIR-CECRI manuscript number: CECRI/PESVC/Pubs./2024-172

We gratefully acknowledge computer time provided by the state of Baden-Württemberg through bwHPC and the DFG through grant no. INST 40/575-1 FUGG (JUSTUS 2 cluster). The work contributes to the research performed at CELEST (Center for Electrochemical Energy Storage Ulm-Karlsruhe).

Received: ((will be filled in by the editorial staff))
Revised: ((will be filled in by the editorial staff))
Published online: ((will be filled in by the editorial staff)

References

[1] H.-D. Lim, B. Lee, Y. Bae, H. Park, Y. Ko, H. Kim, J. Kim, K. Kang, *Chemical Society Reviews* **2017**, *46*, 2873-2888.

[2] S. Guo, L. Zou, M. Sun, Z. Wang, S. Han, B. Chi, J. Pu, J. Li, *ACS Applied Energy Materials* **2020**, *3*, 10015-10022.

[3] G. Liu, H. Chen, L. Xia, S. Wang, L.-X. Ding, D. Li, K. Xiao, S. Dai, H. Wang, *ACS Applied Materials & Interfaces* **2015**, *7*, 22478-22486.

[4] J. Wang, X. Cheng, Z. Li, M. Xu, Y. Lu, S. Liu, Y. Zhang, C. Sun, *ACS Applied Energy Materials* **2018**, *1*, 5557-5566.

[5] K. R. Yoon, K. Shin, J. Park, S.-H. Cho, C. Kim, J.-W. Jung, J. Y. Cheong, H. R. Byon, H. M. Lee, I.-D. Kim, *ACS Nano* **2018**, *12*, 128-139.

[6] Y. Li, J. Lu, *ACS Energy Letters* **2017**, *2*, 1370-1377.

[7] F. Strauss, G. Rousse, M. T. Sougrati, D. A. Dalla Corte, M. Courty, R. Dominko, J.-M. Tarascon, *Inorganic Chemistry* **2016**, *55*, 12775-12782.

[8] P. Barpanda, L. Lander, S. i. Nishimura, A. Yamada, *Advanced Energy Materials* **2018**, *8*, 1703055.

[9] H. Kim, J. Park, I. Park, K. Jin, S. E. Jerng, S. H. Kim, K. T. Nam, K. Kang, *Nature Communications* **2015**, *6*, 1-11.

[10] A. Baby, D. Singh, C. Murugesan, P. Barpanda, *Chemical Communications* **2020**, *56*, 8400-8403.

[11] L. Sharma, R. Gond, B. Senthilkumar, A. Roy, P. Barpanda, *ACS Catalysis* **2019**, *10*, 43-50.

[12] D. Dwibedi, R. Gond, P. Barpanda, *Chemistry of Materials* **2019**, *31*, 7501-7509.

[13] H.-H. Wang, C. Zhang, J. Gao, K. C. Lau, S. T. Plunkett, M. Park, R. Amine, L. A. Curtiss, *Faraday Discussions* **2024**, *248*, 48-59.

[14] P. Seraji, H. Shahbazi, M. K. Ncube, N. Shan, F. Lagunas, I. Papailias, P. Navabi, C. Zhang, A. Jaradat, S. Kadkhodaei, *Nano Energy* **2025**, *134*, 110510.

[15] C. Zhang, S. Wang, T. Yang, N. Shan, S. K. Singh, A. Jaradat, M. K. Ncube, P. Redfern, A. Subramanian, Z. Huang, *Energy Storage Materials* **2023**, *60*, 102844.

[16] J. Lu, Y. Jung Lee, X. Luo, K. Chun Lau, M. Asadi, H.-H. Wang, S. Brombosz, J. Wen, D. Zhai, Z. Chen, *Nature* **2016**, *529*, 377-382.

[17] S. T. Plunkett, A. Kondori, D. Y. Chung, J. Wen, M. Wolfman, S. H. Lapidus, Y. Ren, R. Amine, K. Amine, A. U. Mane, *ACS Energy Letters* **2022**, *7*, 2619-2626.

[18] S. T. Plunkett, C. Zhang, K. C. Lau, M. R. Kephart, J. Wen, D. Y. Chung, D. Phelan, Y. Ren, K. Amine, S. Al-Hallaj, *Nano Energy* **2021**, *90*, 106549.

[19] A. Halder, H.-H. Wang, K. C. Lau, R. S. Assary, J. Lu, S. Vajda, K. Amine, L. A. Curtiss, *ACS Energy Letters* **2018**, *3*, 1105-1109.

[20] A. Halder, A. T. Ngo, X. Luo, H.-H. Wang, J. Wen, P. Abbasi, M. Asadi, C. Zhang, D. Miller, D. Zhang, *The Journal of Physical Chemistry A* **2019**, *123*, 10047-10056.

[21] J. Masa, P. Weide, D. Peeters, I. Sinev, W. Xia, Z. Sun, C. Somsen, M. Muhler, W. Schuhmann, *Advanced Energy Materials* **2016**, *6*, 1502313.

[22] X. Cao, L. Cui, X. Wang, W. Yang, J. Liu, *ChemCatChem* **2018**, *10*, 2826-2832.

[23] Z. Wang, X. Peng, S. Guo, M. Sun, J. Cheng, L. Zou, B. Chi, J. Pu, *ACS Applied Energy Materials* **2021**, *4*, 9376-9383.

[24] Q. Xia, L. Zhao, D. Li, J. Wang, L. Liu, C. Hou, X. Liu, H. Xu, F. Dang, J. Zhang, *Journal of Materials Chemistry A* **2021**, *9*, 19922-19931.

[25] S. Guddehalli Chandrappa, G. Karkera, V. A. Gangadharappa, D. Chen, R. A. Caruso, P. Annigere S, *ACS Applied Energy Materials* **2022**, *5*, 14680-14686.

[26] Y. S. Jeong, J.-B. Park, H.-G. Jung, J. Kim, X. Luo, J. Lu, L. Curtiss, K. Amine, Y.-K. Sun, B. Scrosati, *Nano Letters* **2015**, *15*, 4261-4268.

[27] D. Zhai, H.-H. Wang, J. Yang, K. C. Lau, K. Li, K. Amine, L. A. Curtiss, *Journal of the American Chemical Society* **2013**, *135*, 15364-15372.

[28] M. W. Yuan, C. Y. Nan, Y. Yang, G. B. Sun, H. F. Li, S. L. Ma, *Acs Omega* **2017**, *2*, 4269-4277.

[29] H.-J. Bleif, H. Dachs, *Acta Crystallographica Section A: Crystal Physics, Diffraction, Theoretical and General Crystallography* **1982**, *38*, 470-476.

[30] H. Stehr, *Zeitschrift für Kristallographie-Crystalline Materials* **1967**, *125*, 332-359.

[31] M. García-Mota, M. Bajdich, V. Viswanathan, A. Vojvodic, A. T. Bell, J. K. Nørskov, *The Journal of Physical Chemistry C* **2012**, *116*, 21077-21082.

[32] M. Bajdich, M. García-Mota, A. Vojvodic, J. K. Nørskov, A. T. Bell, *Journal of the American Chemical Society* **2013**, *135*, 13521-13530.

[33] G. Kresse, J. Furthmüller, *Physical Review B* **1996**, *54*, 11169.

[34] G. Kresse, J. Furthmüller, *Computational Materials Science* **1996**, *6*, 15-50.

[35] P. E. Blöchl, *Physical Review B* **1994**, *50*, 17953.

[36] G. Kresse, D. Joubert, *Physical review B* **1999**, *59*, 1758.

[37] J. P. Perdew, K. Burke, M. Ernzerhof, *Physical Review Letters* **1996**, *77*, 3865.

[38] B. Hammer, L. B. Hansen, J. K. Nørskov, *Physical Review B* **1999**, *59*, 7413.

[39] S. Grimme, J. Antony, S. Ehrlich, H. Krieg, *The Journal of Chemical Physics* **2010**, *132*.

[40] J.-D. Chai, M. Head-Gordon, *Physical Chemistry Chemical Physics* **2008**, *10*, 6615-6620.

[41] S. L. Dudarev, G. A. Botton, S. Y. Savrasov, C. Humphreys, A. P. Sutton, *Physical Review B* **1998**, *57*, 1505.

[42] L. Wang, T. Maxisch, G. Ceder, *Physical Review B—Condensed Matter and Materials Physics* **2006**, *73*, 195107.

[43] K. Forster‐Tonigold, J. Kim, J. Bansmann, A. Groß, F. Buchner, *ChemPhysChem* **2021**, *22*, 441-454.

This study investigates sodium cobalt borate (Na_3_CoB_5_O_10_, NCBO) as an oxygen electrocatalyst for non-aqueous lithium-oxygen (Li-O_2_) batteries. NCBO exhibits favorable oxygen evolution reaction activity with a low overpotential and enhanced stability in Li-O_2_ cycling. DFT calculations reveal the significance of tetrahedral Co coordination in optimizing catalytic performance, demonstrating its potential for improving Li-O_2_ battery efficiency.

*Shivaraju Guddehalli Chandrappa,*^a,b,c,d^ Katrin Forster-Tonigold,^d,e^ Vasantha A. Gangadharappa,^a,b^Pavithra Kannan,^a,b^ Kunkanadu R Prakasha,^a,b^ Axel Groß,^d,f^ Maximilian Fichtner,^d,e^ Rachel A. Caruso,^c^ Guruprakash Karkera,^d^ and Annigere S. Prakash*^a,b^*

Cobalt Borate Complex with Tetrahedrally Coordinated Co^2+^- Promotes Lithium Superoxide formation in Li-O_2_ Batteries

Supporting Information

Cobalt Borate Complex with Tetrahedrally Coordinated Co^2+^- Promotes Lithium Superoxide formation in Li-O_2_ Batteries

*Shivaraju Guddehalli Chandrappa,*^a,b,c,d^Katrin Forster-Tonigold,^d,e^Vasantha A. Gangadharappa,^a,b^Pavithra Kannan,^a,b^ Kunkanadu R Prakasha,^a,b^ Axel Groß,^d,f^ Maximilian Fichtner,^d,e^ Rachel A. Caruso,^c^ Guruprakash Karkera,^d^ and Annigere S. Prakash*^a,b^*

**Table S1.** The structural parameters determined by Rietveld refinement for NCBO powder X-ray diffraction pattern.

| **Atom** | **Wyckoff site** | ***x*** | ***y*** | ***z*** | **Biso (Å^2^)** |
| --- | --- | --- | --- | --- | --- |
| Na1 | 4e | 0.9814(1) | 0.0711 | 0.1348(2) | 1.45(1) |
| Na2 | 4e | 0.3656(3) | 0.7410(7) | -0.4402(2) | 2.23(5) |
| Na3 | 4e | 0.2395(3) | 0.2367(3) | -0.1818(6) | 0.79(4) |
| Co | 4e | 0.4955(3) | 0.0935(1) | 0.1170(3) | 0.93(3) |
| B1 | 4e | 0.7146(1) | 0.2060(8) | -0.0654 | 0.89 |
| B2 | 4e | 0.0782(5) | 0.1063(2) | -0.2224(2) | 1.02(6) |
| B3 | 4e | 0.0632(3) | 0.1086(1) | -0.4358(6) | 1.15(8) |
| B4 | 4e | 0.2236(8) | 0.1688(2) | 0.3867(7) | 0.68(9) |
| B5 | 4e | 0.8250(1) | 0.0785(1) | 0.4968(1) | 0.83(9) |
| O1 | 4e | 0.3945 | 0.3694(7) | -0.0599(2) | 0.84(2) |
| O2 | 4e | 0.7269(2) | 0.1264(2) | 0.0023(1) | 0.84(1) |
| O3 | 4e | 0.6496(2) | 0.0519(5) | 0.3837(8) | 0.84 |
| O4 | 4e | 0.2142 | 0.1300(9) | -0.2704(9) | 0.84(9) |
| O5 | 4e | 0.2145(3) | 0.1401(8) | -0.5753(1) | 0.84(7) |
| O6 | 4e | 0.2154 | 0.0493(2) | -0.0863(6) | 0.84(2) |
| O7 | 4e | 0.1422(3) | 0.2390(8) | -0.4502(5) | 0.84(5) |
| O8 | 4e | 1.0724(3) | -0.0488(5) | 0.3282(4) | 0.84(2) |
| O9 | 4e | 0.3126(5) | 0.1769(1) | 0.2035(7) | 0.84 |
| O10 | 4e | -0.2627 | 0.2286(7) | -0.1819 | 0.83(9) |
|  |  |  |  |  |  |
| *a* = 6.6448(7) Å, *b* = 18.2061(2) Å, *c* = 7.8091(8) Å, α = γ = 90°, β = 114.81°,  Space group = P2_1_/n, Χ^2^ = 2.93 | | | | | |

**Table S2.** Values of the R_1_ and R_3_ obtained by simulated data of the impedance spectrum of NCBO.

| **Sample** | **R_1_/ ohm** | **R_3_/ ohm** |
| --- | --- | --- |
| Pristine | 24.4 | 69.7 |
| Discharged | 72.3 | 558.8 |
| Charged | 117.8 | 303.4 |

**Figure S1.** **(a)** XPS survey scan and **(b)** high-resolution XPS spectrum of the O 1s region for NCBO.

**Figure 0.1.** **(a)** XPS survey scan and high-resolution XPS spectra of **(b)** Co 2p, **(c)** B 1s, and (**d)** O1s regions for NCBO.

**Figure 0.2.** **(a)** XPS survey scan and high-resolution XPS spectra of **(b)** Co 2p, **(c)** B 1s, and (**d)** O1s regions for NCBO.

**Figure 0.3.** **(a)** XPS survey scan and high-resolution XPS spectra of **(b)** Co 2p, **(c)** B 1s, and (**d)** O1s regions for NCBO.

**Figure 0.4.** **(a)** XPS survey scan and high-resolution XPS spectra of **(b)** Co 2p, **(c)** B 1s, and (**d)** O1s regions for NCBO.

**Figure 0.5.** **(a)** XPS survey scan and high-resolution XPS spectra of **(b)** Co 2p, **(c)** B 1s, and (**d)** O1s regions for NCBO.

**Figure 0.6.** **(a)** XPS survey scan and high-resolution XPS spectra of **(b)** Co 2p, **(c)** B 1s, and (**d)** O1s regions for NCBO.

**Figure 0.7.** **(a)** XPS survey scan and high-resolution XPS spectra of **(b)** Co 2p, **(c)** B 1s, and (**d)** O1s regions for NCBO.

**Figure 0.8.** **(a)** XPS survey scan and high-resolution XPS spectra of **(b)** Co 2p, **(c)** B 1s, and (**d)** O1s regions for NCBO.

**Figure 0.9.** **(a)** XPS survey scan and high-resolution XPS spectra of **(b)** Co 2p, **(c)** B 1s, and (**d)** O1s regions for NCBO.

**Figure 0.10.** **(a)** XPS survey scan and high-resolution XPS spectra of **(b)** Co 2p, **(c)** B 1s, and (**d)** O1s regions for NCBO.

**Figure 0.11.** **(a)** XPS survey scan and high-resolution XPS spectra of **(b)** Co 2p, **(c)** B 1s, and (**d)** O1s regions for NCBO.

**Figure 0.12.** **(a)** XPS survey scan and high-resolution XPS spectra of **(b)** Co 2p, **(c)** B 1s, and (**d)** O1s regions for NCBO.

**Figure 0.13.** **(a)** XPS survey scan and high-resolution XPS spectra of **(b)** Co 2p, **(c)** B 1s, and (**d)** O1s regions for NCBO.

**Figure 0.14.** **(a)** XPS survey scan and high-resolution XPS spectra of **(b)** Co 2p, **(c)** B 1s, and (**d)** O1s regions for NCBO.

**Figure 0.15.** **(a)** XPS survey scan and high-resolution XPS spectra of **(b)** Co 2p, **(c)** B 1s, and (**d)** O1s regions for NCBO.

**Figure 0.16.** **(a)** XPS survey scan and high-resolution XPS spectra of **(b)** Co 2p, **(c)** B 1s, and (**d)** O1s regions for NCBO.

**Figure 0.17.** **(a)** XPS survey scan and high-resolution XPS spectra of **(b)** Co 2p, **(c)** B 1s, and (**d)** O1s regions for NCBO.

**Figure 0.18.** **(a)** XPS survey scan and high-resolution XPS spectra of **(b)** Co 2p, **(c)** B 1s, and (**d)** O1s regions for NCBO.

**Figure 0.19.** **(a)** XPS survey scan and high-resolution XPS spectra of **(b)** Co 2p, **(c)** B 1s, and (**d)** O1s regions for NCBO.

**Figure 0.20.** **(a)** XPS survey scan and high-resolution XPS spectra of **(b)** Co 2p, **(c)** B 1s, and (**d)** O1s regions for NCBO.

**Figure 0.21.** **(a)** XPS survey scan and high-resolution XPS spectra of **(b)** Co 2p, **(c)** B 1s, and (**d)** O1s regions for NCBO.

**Figure 0.22.** **(a)** XPS survey scan and high-resolution XPS spectra of **(b)** Co 2p, **(c)** B 1s, and (**d)** O1s regions for NCBO.

**Figure 0.23.** **(a)** XPS survey scan and high-resolution XPS spectra of **(b)** Co 2p, **(c)** B 1s, and (**d)** O1s regions for NCBO.

**Figure 0.24.** **(a)** XPS survey scan and high-resolution XPS spectra of **(b)** Co 2p, **(c)** B 1s, and (**d)** O1s regions for NCBO.

**Figure 0.25.** **(a)** XPS survey scan and high-resolution XPS spectra of **(b)** Co 2p, **(c)** B 1s, and (**d)** O1s regions for NCBO.

**Figure 0.26.** **(a)** XPS survey scan and high-resolution XPS spectra of **(b)** Co 2p, **(c)** B 1s, and (**d)** O1s regions for NCBO.

**Figure 0.27.** **(a)** XPS survey scan and high-resolution XPS spectra of **(b)** Co 2p, **(c)** B 1s, and (**d)** O1s regions for NCBO.

**Figure 0.28.** **(a)** XPS survey scan and high-resolution XPS spectra of **(b)** Co 2p, **(c)** B 1s, and (**d)** O1s regions for NCBO.

**Figure 0.29.** **(a)** XPS survey scan and high-resolution XPS spectra of **(b)** Co 2p, **(c)** B 1s, and (**d)** O1s regions for NCBO.

**Figure 0.30.** **(a)** XPS survey scan and high-resolution XPS spectra of **(b)** Co 2p, **(c)** B 1s, and (**d)** O1s regions for NCBO.

**Figure 0.31.** **(a)** XPS survey scan and high-resolution XPS spectra of **(b)** Co 2p, **(c)** B 1s, and (**d)** O1s regions for NCBO.

**Figure S2.** The CP studies of the NCBO catalyst.

**Ex-situ XPS.**

**Figure S3.** Ex-situ XPS spectra of pristine and cycled electrodes. (a) Co 2p and (b) Li 1s.

Furthermore, ex-situ XPS analysis of the cycled NCBO cathodes was carried out. **Figure S3a** shows the Co 2p spectra of the pristine and cycled cathodes. In the case of the discharged electrode, no clear Co 2p spectra were observed, which might be due to the accumulation of discharge products (Li_2_O_2_/LiO_2_) on the cathode surface. In contrast, the charged cathode exhibited a slight shift of the Co 2p peaks toward higher binding energy, indicating a minor structural change in the cobalt environment during cycling.

**Figure S3b** presents the Li 1s spectra of the pristine and cycled cathodes**.** The pristine electrode does not show a Li 1s peak, whereas the discharged sample exhibits a distinct Li 1s peak at a binding energy of 55.5 eV, confirming the formation of Li_2_O_2_/LiO_2_. Notably, the Li 1s peak is absent in the charged electrode, suggesting complete dissociation of Li_2_O_2_/LiO_2_ from the surface of the NCBO cathode.


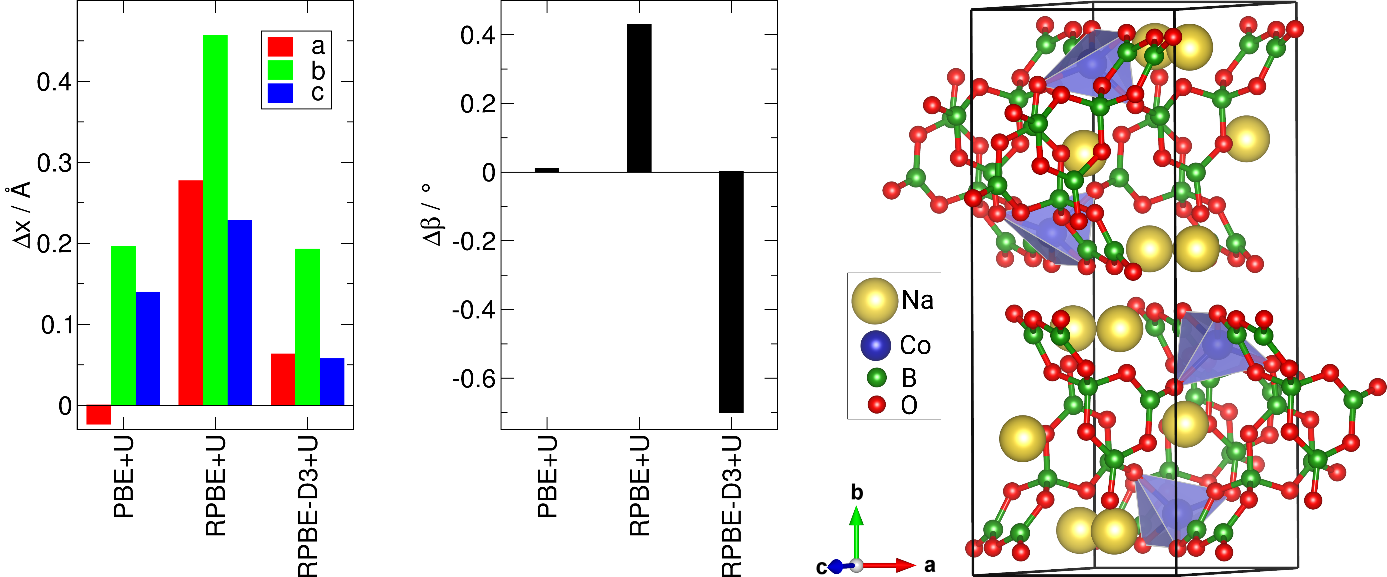


**Figure S4.** (a) Deviation of the calculated lengths (Δx=|**x**^calc^|-|**x**^exp^|; x={a,b,c}) and (b) angle β (Δβ) of the lattice vectors **a**, **b** and **c** of the NCBO crystal from experiment.

**Table S3.** Parameters of the crystal structure of NCBO as calculated by different functionals (PBE+U, RPBE+U, RPBE-D3+U with U=3.5 eV, if not denoted otherwise) and measured in experiment.

|  | experiment | PBE+U | RPBE+U | RPBE-D3+U(U=3.5 eV) | RPBE-D3+U(U=2.5 eV) |
| --- | --- | --- | --- | --- | --- |
| a / Å | 6.6448(7) | 6.6218 | 6.9221 | 6.7082 | 6.7062 |
| b / Å | 18.2061(2) | 18.4026 | 18.6625 | 18.3990 | 18.3995 |
| c / Å | 7.8091(8) | 7.9489 | 8.0372 | 7.8667 | 7.8723 |
| α=γ / ° | 90 | 90 | 90 | 90 | 90 |
| β / ° | 114.81 | 114.82 | 115.24 | 114.11 | 114.17 |

The PBE, RPBE and the dispersion corrected RPBE functional (RPBE-D3), all augmented with a Hubbard-like correction for the correlated d electrons of Co (furtheron denoted as “+U”), were used to calculate the crystal structure of NCBO. Despite its inability to account for dispersive interactions, the PBE+U method describes the crystal structure of NCBO remarkably well. In contrast, the RPBE functional in conjunction with the Hubbard correction clearly overestimates the structural parameters. If in addition dispersive interactions are taken into account, the RPBE-D3+U method calculates values very close to those experimentally obtained. Finally, we would like to note, that the value of the Hubbard correction (U=2.5 eV vs. 3.5 eV) on top of the dispersion corrected RPBE functional has hardly any impact on the structural parameters of the NCBO crystal (|x(U=3.5 eV) – x(U=2.5 eV)|^max^=5.68×10^-3^ Å, |β(U=3.5 eV) – β(U=2.5 eV)|=0.06°.

**
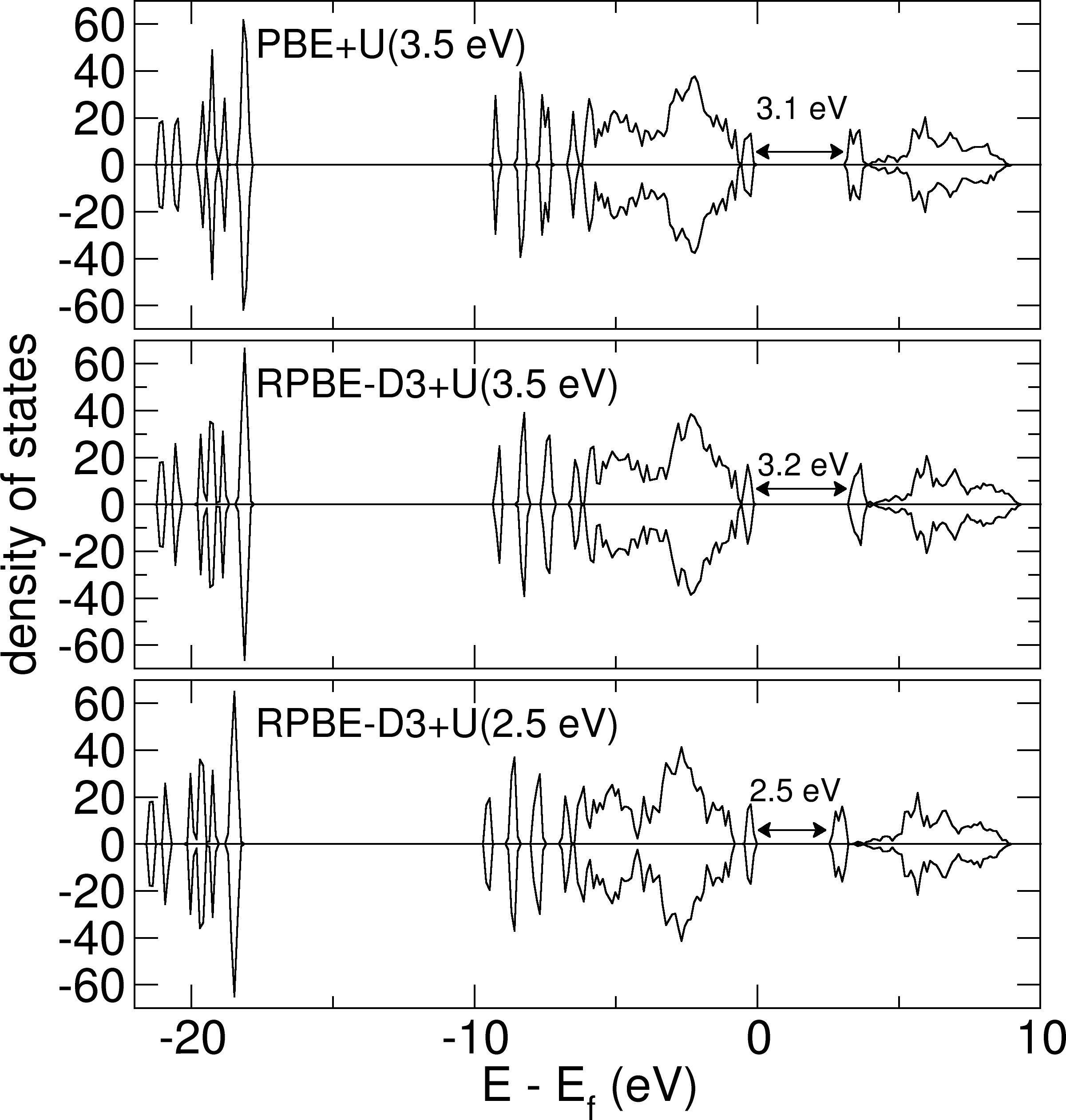
**

**Figure S5.** Density of states of the NCBO crystal calculated with the PBE+U and RPBE-D3+U functional as labelled.


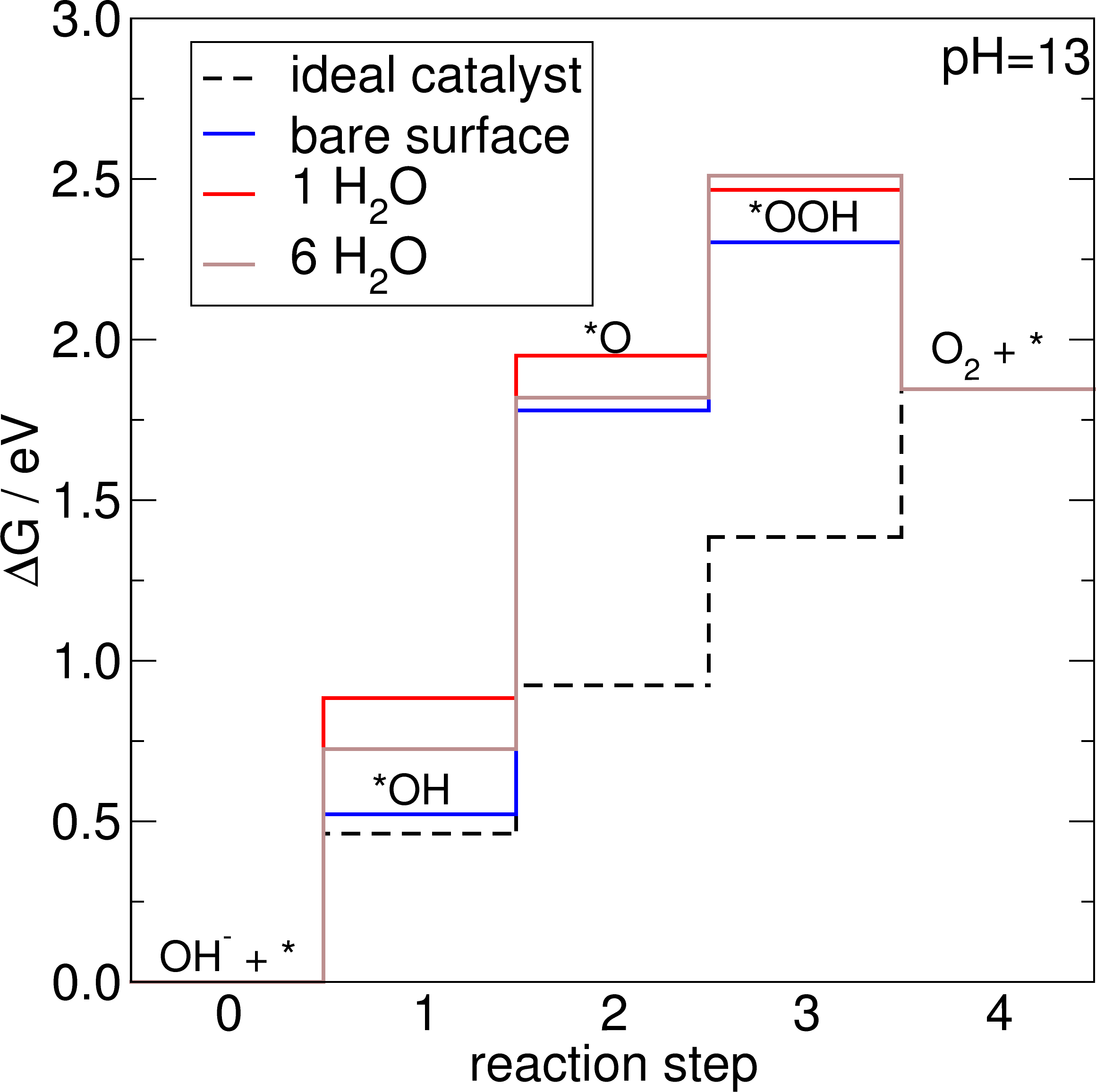

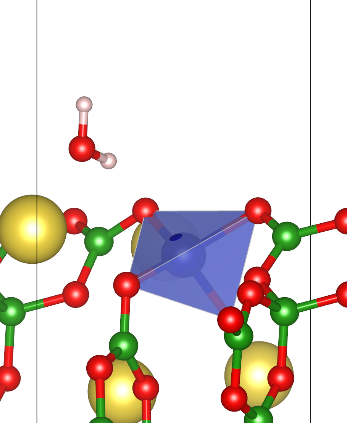

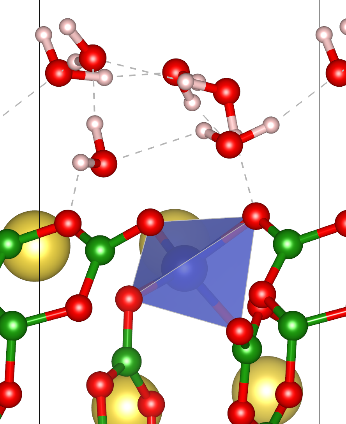


**Figure S6**. Reaction energies $\text{∆G(}j\text{)}=\sum_{i}^{j} \text{∆}\text{G}_{i}$, with *i*=1…4, for the alkaline OER reaction steps calculated with the PBE+U method at (010) surfaces with one (middle panel) or 6 (right panel) co-adsorbed water molecules per surface unit cell.

Calculations of the alkaline OER mechanism can be related to the acidic mechanism (see Qiuhua Liang *et al.* *J. Phys. Energy* **2021**, 3, 026001). Hence the reaction energies (∆G_i_) are calculated according to:

∆G_1_ = E(OH*) – E(*) – E(H_2_O) + 1/2 E(H_2_) + (∆ZPE − T∆S)_1_ – eU_SHE_ – k_B_T x ln(10) x pH (6)
∆G_2_ = E(O*) – E(OH*) + 1/2 E(H_2_) + (∆ZPE − T∆S)_2_ – eU_SHE_ – k_B_T x ln(10) x pH (7)
∆G_3_ = E(OOH*) – E(O*) – E(H_2_O) + 1/2 E(H_2_) + (∆ZPE − T∆S)_3_ – eU_SHE_ – k_B_T x ln(10) x pH (8)
∆G_4_ = 4 x (1.23 eV – eU_SHE_ – k_B_T x ln(10) x pH) – (∆G_1_+∆G_2_+∆G_3_) (9)

Here E(X) or E(X*) denote the total DFT energies of the adsorption complexes (X*), the bare surface (*) or the isolated molecules (X). The changes in the zero point energies and entropy (ΔZPE-TΔS) have been taken from literature (Kim, H., Park, J., Park, I. *et al.* Coordination tuning of cobalt phosphates towards efficient water oxidation catalyst. *Nat Commun* **6**, 8253 (2015). https://doi.org/10.1038/ncomms9253).

Finally, the overpotential, η, is determined according to the following equation.

η=[max(ΔG_1_, ΔG_2_, ΔG_3_, ΔG_4_) – eU_0_ + k_B_T x ln(10) x pH] / e (10)
